# The Functional Connectome of Speech Control

**DOI:** 10.1371/journal.pbio.1002209

**Published:** 2015-07-23

**Authors:** Stefan Fuertinger, Barry Horwitz, Kristina Simonyan

**Affiliations:** 1 Department of Neurology, Icahn School of Medicine at Mount Sinai, New York, New York, United States of America; 2 Brain Imaging and Modeling Section, National Institute on Deafness and Other Communication Disorders, National Institutes of Health, Bethesda, Maryland, United States of America; 3 Department of Otolaryngology, Icahn School of Medicine at Mount Sinai, New York, New York, United States of America; Research Center Jülich, GERMANY

## Abstract

In the past few years, several studies have been directed to understanding the complexity of functional interactions between different brain regions during various human behaviors. Among these, neuroimaging research installed the notion that speech and language require an orchestration of brain regions for comprehension, planning, and integration of a heard sound with a spoken word. However, these studies have been largely limited to mapping the neural correlates of separate speech elements and examining distinct cortical or subcortical circuits involved in different aspects of speech control. As a result, the complexity of the brain network machinery controlling speech and language remained largely unknown. Using graph theoretical analysis of functional MRI (fMRI) data in healthy subjects, we quantified the large-scale speech network topology by constructing functional brain networks of increasing hierarchy from the resting state to motor output of meaningless syllables to complex production of real-life speech as well as compared to non-speech-related sequential finger tapping and pure tone discrimination networks. We identified a segregated network of highly connected local neural communities (hubs) in the primary sensorimotor and parietal regions, which formed a commonly shared core hub network across the examined conditions, with the left area 4p playing an important role in speech network organization. These sensorimotor core hubs exhibited features of flexible hubs based on their participation in several functional domains across different networks and ability to adaptively switch long-range functional connectivity depending on task content, resulting in a distinct community structure of each examined network. Specifically, compared to other tasks, speech production was characterized by the formation of six distinct neural communities with specialized recruitment of the prefrontal cortex, insula, putamen, and thalamus, which collectively forged the formation of the functional speech connectome. In addition, the observed capacity of the primary sensorimotor cortex to exhibit operational heterogeneity challenged the established concept of unimodality of this region.

## Abstract 2

This study uses graph theory to analyze functional MRI data recorded from speakers as they produce single syllables or whole sentences, revealing the complexity of the brain network machinery that controls speech and language.

## Author Summary

Speech production is a complex process that requires the orchestration of multiple brain regions. However, our current understanding of the large-scale neural architecture during speaking remains scant, as research has mostly focused on examining distinct brain circuits involved in distinct aspects of speech control. Here, we performed graph theoretical analyses of functional MRI data acquired from healthy subjects in order to reveal how brain regions relate to one another while speaking. We constructed functional brain networks of increasing hierarchy from rest to simple vocal motor output to the production of real-life speech, and compared these to nonspeech control tasks such as finger tapping and pure tone discrimination. We discovered a specialized network of densely connected sensorimotor regions, which formed a common processing core across all conditions. Specifically, the primary sensorimotor cortex participated in multiple functional domains across different networks and modulated long-range connections depending on task content, which challenges the established concept of low-order unimodal function of this region. Compared to other tasks, speech production was characterized by the formation of six distinct neural communities with specialized recruitment of the prefrontal cortex, insula, putamen, and thalamus, which collectively formed the functional speech connectome.

## Introduction

Extensive neuroimaging research over the past two decades installed the notion that speech and language require an orchestration between several brain regions for comprehension, planning, and integration of a heard sound with a spoken word [1–6]. However, studies investigating brain networks of speech and language control have been largely limited to the examination of distinct cortical and subcortical circuits involved in a range of speech controlling components, such as speech motor output [7–13], verbal fluency [14–16], phonological and semantic processing [17–21], verbal and tonal working memory [22–26], speech monitoring and discrimination [27–29], neural synchronization [30,31], and integration [32–38]. Moreover, the majority of these studies were directed toward mapping the neural correlates of separate speech elements, such as production of meaningless syllable sequences or single words [7,13,39–46], with only a handful of studies examining real-life speech production [6,9,38,47,48]. As a result, our understanding of the complexity of brain network machinery controlling speech and language is very limited. One particularly significant and outstanding question concerns the large-scale architecture, interactions, and functional specialization of brain regions within the speech network for shaping the production of spoken language.

Here, we applied graph theoretical analysis [49–52] to functional MRI (fMRI) data of healthy adult individuals during the resting state, production of meaningless syllables as a motor task relevant to speaking but with minimal linguistic meaning, and production of grammatically correct, meaningful real-life English sentences in order to examine functional networks of increasing hierarchy and to quantify the intermediate steps in the formation of the speech production network. To further delineate speech network characteristics and community-based architecture, we conducted a follow up study to investigate the formation of nodal communities across all examined conditions, as well as in comparison with the modular structure of functional networks during the performance of a nonlinguistic task (i.e., auditory temporal discrimination of pure tones) and a simple nonspeech motor task (i.e., sequential finger tapping). In the first experiment, we hypothesized that the speech production network (SPN), compared to the resting state (RSN) and syllable production (SylPN) networks, would exhibit enhanced functional segregation with densely interconnected local communities (hubs) centered on the sensorimotor cortex. Importantly, functional specialization of these sensorimotor hubs during speech production would ensure the distinct and unique recruitment of the multimodal integrative cortical regions, such as the prefrontal and inferior parietal cortices, into the SPN but not the RSN or SylPN. In the second experiment, we hypothesized that, while the functional network during each condition (i.e., resting state, syllable production, speech production, finger tapping, and auditory discrimination) would be characterized by a distinct community-based structure, the emergence of specialized processing communities and refined modular architecture of SPN would uniquely reflect the complexity of network configuration for speech production.

## Results

### Experiment 1: Complexity of the Functional Connectome from the Resting State to Meaningless Syllable Production to Meaningful Speaking

#### Global network metrics

Group-averaged SPN, SylPN, and RSN were computed in 14 healthy right-handed monolingual English speakers as reported earlier [53,54]. All graphs showed small world characteristics with their respective small world indices being larger than one over the established connection density range of 60%–78%. Small-worldness was more pronounced for SPN (all σ > 1.5) and SylPN (all σ > 1.7) than RSN (all σ > 1.01).

Global clustering coefficient and global efficiency were calculated for the RSN, SylPN, and SPN to obtain a first estimate of the degree of segregation and integration of each network. Compared to random networks, all real networks showed high-clustering coefficient (SPN 0.50; SylPN 0.56; RSN 0.33 versus random network 0.32) and high global efficiency (normalized *E*_*glob*_ = 0.9998 SPN, 0.9899 SylPN, 0.9992 RSN). Collectively, the computed graph metrics showed the topological stability of the RSN, SylPN, and SPN across connection densities (see Results in S1 Text and S1 Fig). Following the rationale that brain networks prefer a lower wiring cost at a comparable adaptive value [55,56], we focused our comparative analysis of the SPN and RSN as well as the SPN and SylPN on group-averaged networks at their minimum density of 60%.

#### Nodal attributes of SPN and RSN

**Fig 1:**
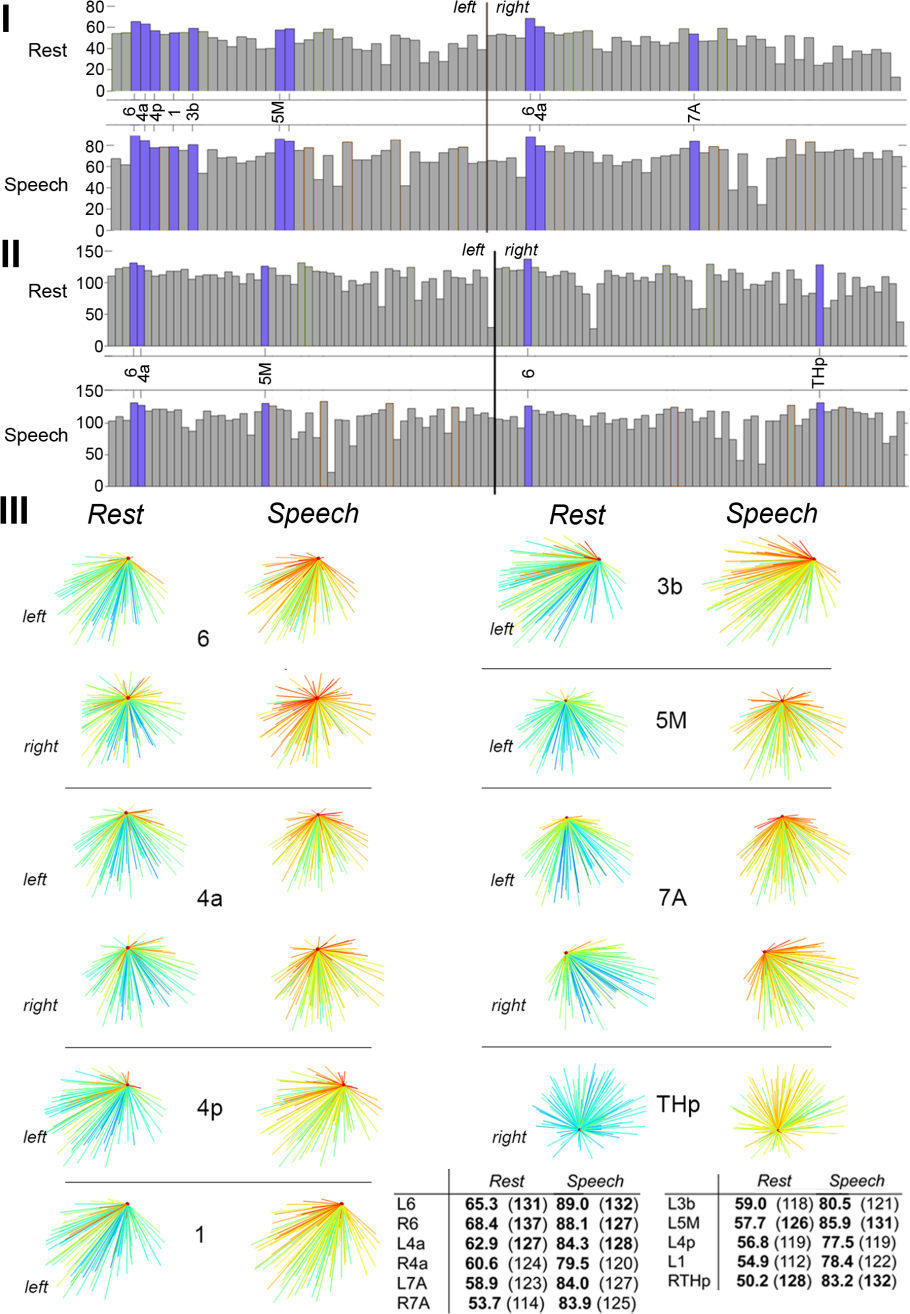
Topology of shared high-degree and high-strength hubs in the group-averaged RSN and SPN at the minimum density of 60%. (I) Bar charts show strength values of the top 30% strongest nodes (normalized *s*_*i*_ ranges 0.9–1.0 and 0.7–0.89 in Fig 4) in both the RSN and SPN. Blue bars highlight nodes that are strength hubs in both the RSN and SPN. (II) Bar charts of the same format show shared degree hubs of the RSN and SPN among the top 30% most interconnected nodes (normalized *k*_*i*_ ranges 0.9–1.0 and 0.7–0.89 in Fig 5). (III) The panel demonstrated the strength and degree of hubs shared by the RSN and SPN. All hubs showed a pronounced increase in strength (sum of connected edge weights) from the resting state to speech production (a shift from blue to red connections) with only a moderate, if at all, increase in degree (number of connections). The table provides quantitative measures of nodal values of strength and degree (in parenthesis). Bold numbers indicate that a node is a hub with respect to the respective metric. The 3-D graphs were rendered using Mayavi [57]. Abbreviations: 1 = area 1; 3a = area 3a; 4a/4p = anterior/posterior part of area 4; 5M = area 5M; 6 = area 6; 7A = area 7A; THp = parietal part of the thalamus. The corresponding data are publicly available at http://figshare.com/articles/The_Functional_Connectome_of_Speech_Control/1431873

**Fig 2:**
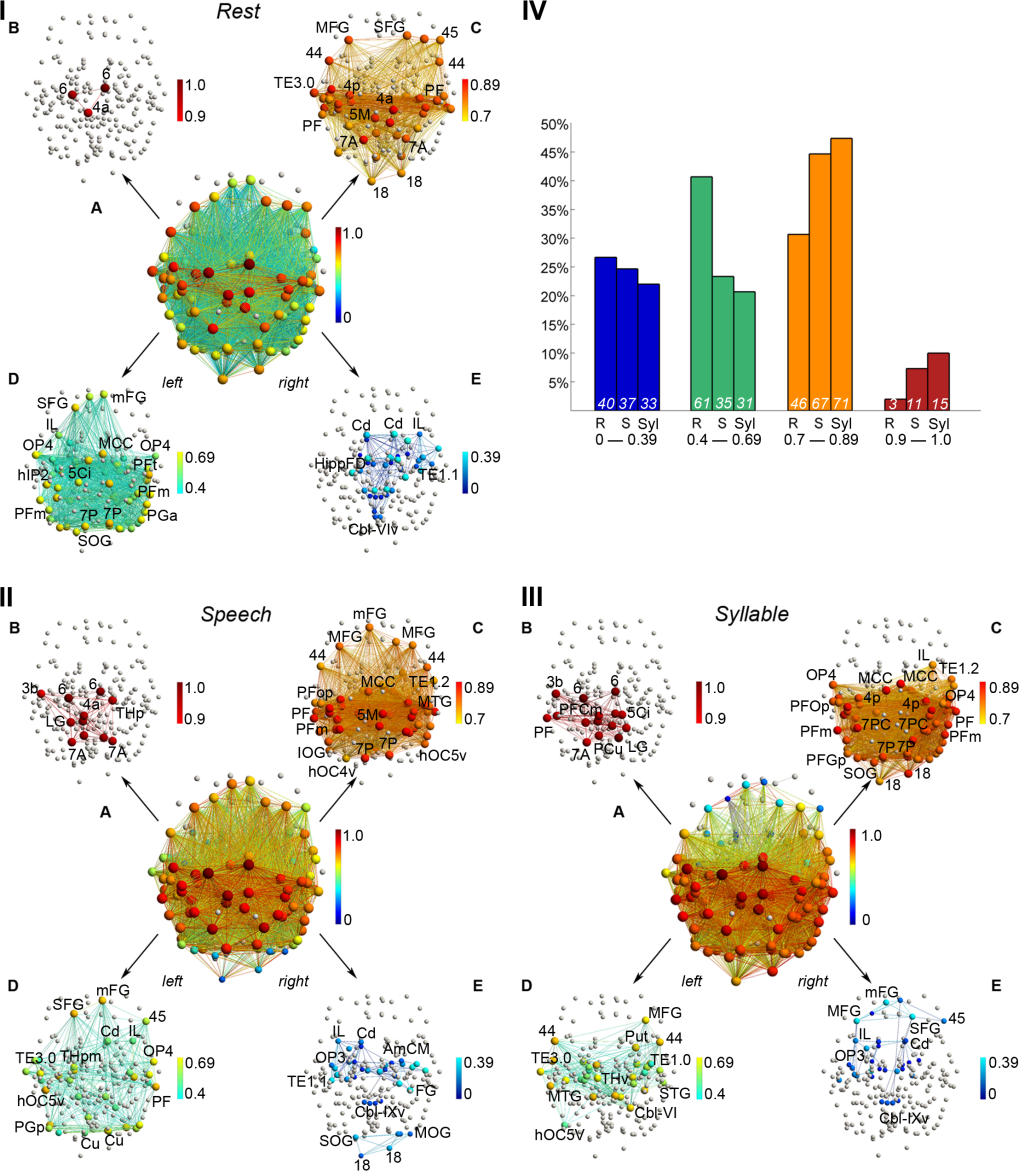
Nodal strength of the group-averaged networks at the minimum density of 60%. (I,II,III) In the 3-D network visualizations, edge color represents link weight, nodal color corresponds to normalized strength, and nodal size illustrates degree. (IA–E, IIA–E, IIIA–E) Nodes that were removed by the employed elimination strategy are shown in gray. In all networks, strength was normalized to the interval [0 (dark blue)– 1 (dark red)] and split up into four distinct ranges; only nodes with normalized strength in the respective ranges are color-coded. (IV) Bar charts illustrate the proportion of nodes in the respective strength intervals relative to the total number of nodes in the networks. Numbers at the bottom of the bars are node counts for the R = resting state, S = speech production, and Syl = syllable production networks in the corresponding intervals. The 3-D networks were visualized with the BrainNet Viewer (http://www.nitrc.org/projects/bnv/). Abbreviations: 1 = area 1; 3a/3b = areas 3a/3b; 44 = area 44; 45 = area 45; 4a/4p = anterior/posterior part of area 4; 5Ci = area 5Ci; 5M = area 5M; 6 = area 6; 7A/7P = area 7A/7P; 18 = area 18; AmCM = subdivision CM of the amygdala; Cbl-V/VIv/IX/IXv = cerebellar lobules V/VIv/IX/IXv; Cd = caudate nucleus; Cu = cuneus; FG = fusiform gyrus; hIP1-3 = areas hIP1-3; HippFD/SUB = hippocampal subdivisions FD/SUB; hOC4v/hOC5v = ventral parts of areas hOC4/hOC5; IL = insula; IOG/MOG/SOG = inferior/middle/superior occipital gyrus; LG = lingual gyrus; MCC = middle cingulate cortex; MTG = middle temporal gyrus; OP1-4 = operculum; PF/PFm/PFop/PFt/PGa/PGp = areas PF/PFm/PFop/PFt/PGa/PGp in the inferior parietal cortex; SMG/MFG/mFG = superior/middle/medial frontal gyrus; TE1.1–3.0 = areas TE1.1–3.0; THp = parietal part of the thalamus. All connectivity matrices are publicly available at http://figshare.com/articles/The_Functional_Connectome_of_Speech_Control/1431873; the codes used to transform the fMRI data to networks can be found at http://research.mssm.edu/simonyanlab/analytical-tools/.

*Shared network hubs*. The functional influence of nodes within each network was quantified by computing the nodal degree k_i_ (the number of edges a node participates in) and the nodal strength s_i_ (the sum of edge weights connected to the node). Among the top 30% strongest network nodes (normalized s_i_ ≥ 0.7) in the SPN and RSN (total of 82 nodes), both networks shared ten high-strength hubs, including the premotor cortex (bilateral area 6), primary motor cortex (bilateral area 4a and left area 4p), primary somatosensory cortex (left areas 3b and 1), and parietal cortex (bilateral area 7A and left area 5M) (Fig 1I and 1II, Table 1). Out of these 10 shared hubs, 7 SPN hubs and 3 RSN hubs were located within the strongest 10% of nodes (normalized s_i_ = 0.9−1.0), while 3 SPN hubs and 7 RSN hubs were found within the top 20%–30% network strength (normalized s_i_ = 0.7–0.89) (Fig 2IB and 2IC, 2IIB and 2IIC). Strength values were significantly higher for all SPN hubs compared to the RSN hubs (all *p* ≤ 0.049, corrected), except for the hub in the bilateral area 4a, which did not show significant differences between the SPN and RSN (*p* ≥ 0.06).

**Table 1:** Top 30% shared and distinct hubs and high-influence nodes in the RSN and SPN (normalized strength/degree range from 0.7 to 1.0).

Shared hubs in RSN and SPN	Distinct nodes in SPN but not RSN	Distinct nodes in RSN but not SPN
*Strength*		
Premotor cortex	Inferior parietal lobule	Inferior parietal lobule
L Area 6 ‒ RSN 65.3; SPN 89.0	L Angular gyrus (area PGa) – 69.5	L Angular gyrus (area PGp) – 47.5
R Area 6 ‒ RSN 68.4; SPN 88.1	R Angular gyrus (area PGa) – 69.9	R Supramarginal gyrus (area PFop) – 51.3
	L Supramarginal gyrus (area PFm) – 69.1	
	R Supramarginal gyrus (area PFm) – 66.5	
Primary motor cortex	Intraparietal sulcus	Auditory cortex
L Area 4a ‒ RSN 63.0; SPN 84.3	L Areas hIP1, 3–77.5 ± 1.1	L Area TE3.0–55.1
R Area 4a ‒ RSN 60.6; SPN 79.5	R Areas hIP1, 2, 3 ‒ 71.7 ± 4.1	
L Area 4p ‒ RSN 56.8; SPN 77.6		
Primary somatosensory cortex	Parietal cortex	Inferior temporal gyrus
L Area 3b—RSN 59.0; SPN 80.5	L Area 5Ci – 72.7	L – 49.1
L Area 1 ‒ RSN 54.9; SPN 78.4	R Area 5Ci – 71.0	R – 48.6
	L Area 7P – 75.2	
	R Area 7P – 72.9	
Parietal cortex	Auditory cortex	Cuneus
L Area 7A ‒ RSN 58.7; SPN 84.0	R Area TE1.2–62.2	L ‒ 49.7
R Area 7A ‒ RSN 53.7; SPN 83.9		R ‒ 45.4
L Area 5M ‒ RSN 57.5; SPN 85.9		
	Cingulate cortex	Occipital cortex
	L Middle cingulate cortex ‒ 70.3	L Area 18 ‒ 47.3
	R Middle cingulate cortex ‒ 62.2	R Area 18 ‒ 48.7
	L Posterior cingulate cortex – 75.1	
	R Posterior cingulate cortex – 68.6	
	Operculum	
	R Area OP1–63.5	
	Insula	
	R Area Ig1 ‒ 70.2	
	R Area Id1–61.9	
	Cerebellum	
	L Lobule VI ‒ 64.3	
	R Lobule VI ‒ 69.3	
	Thalamus	
	L Prefrontal, Somatosensory, Temporal subdivisions – 67.0 ± 5.2	
	R Prefrontal, Premotor, Motor, Somatosensory, Temporal, Visual subdivisions – 72.9 ± 3.0	
	Putamen	
	R ‒ 62.0	
*Degree*		
Premotor cortex	Primary somatosensory cortex	Operculum
L Area 6 ‒ RSN 131; SPN 132	R Area 1 ‒ 113	L OP4 ‒ 105
R Area 6 ‒ RSN 137; SPN 127		
Primary motor cortex	Operculum	Occipital cortex
L Area 4a ‒ RSN 127; SPN 128	R Areas OP1, OP4–100 ± 7.1	L Area 18–115
R Area 4a ‒ RSN 124; SPN 130		R Areas 17, 18–111 ± 13.4
Parietal cortex	Insula	Cuneus
L Area 5M ‒ RSN 126; SPN 131	R Area Id1 ‒ 111	L ‒ 119
Thalamus	Intraparietal sulcus	Inferior temporal gyrus
R Parietal subdivision ‒ RSN 128; SPN 132	R Areas hIP1, 2, 3–112 ± 4.9	L ‒ 118
		R ‒ 103
	Auditory cortex	Inferior frontal gyrus
	R Areas TE1.1, 1.2–105 ± 4.2	R Area 45 ‒ 120
	Middle temporal gyrus	
	R ‒ 119	
	Posterior cingulate cortex	
	L ‒ 121	
	Putamen	
	R ‒ 106	
	Globus pallidus, external segment	
	R ‒ 93	
	Thalamus	
	L Prefrontal subdivision ‒ 108	
	R Prefrontal subdivision ‒ 118	

Left column: Shared high-strength and high-degree hubs in the RSN and SPN networks. Middle column: Distinct high-strength and high-degree nodes in the SPN but not the RSN. Right column: Distinct high-strength and high-degree nodes in the RSN but not the SPN. Values indicate the strength or degree of the node, respectively. Mean ± standard deviation (SD) is given for those nodes that are in similar cytoarchitectonic regions. R–right; L–left.

**Fig 3:**
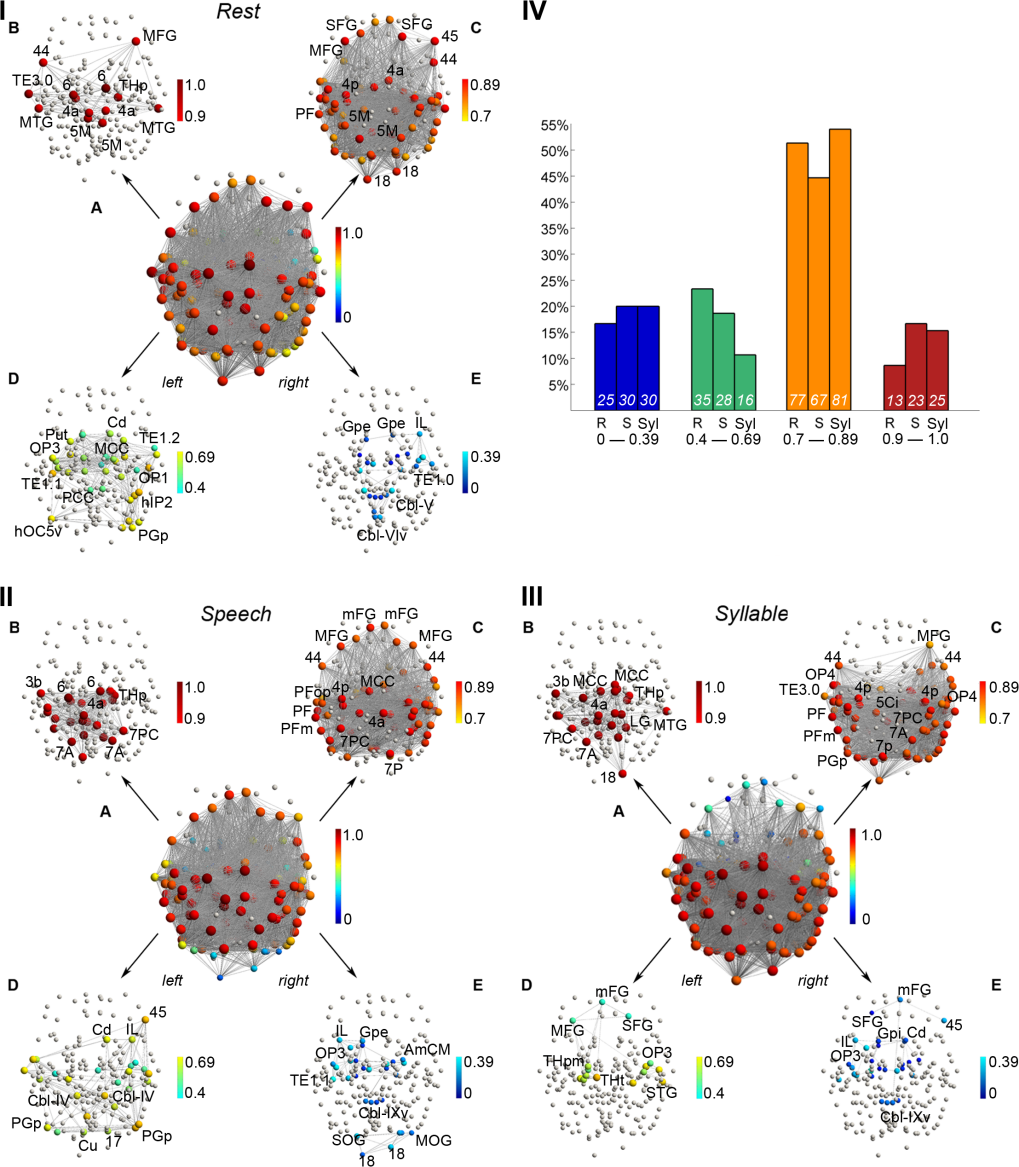
Nodal degree of the group averaged networks at the minimum density of 60%. (I,II,III) In the 3-D network visualizations, both nodal color and size illustrate (normalized) degree. (IA–E, IIA–E, IIIA-E) Nodes that were removed by the employed elimination strategy are shown in gray. In all networks, degree was normalized to the interval [0 (dark blue) – 1 (dark red)] and split up into four distinct ranges; only nodes with normalized degree in the respective range are color-coded. (IV) Bar charts illustrate the proportion of nodes in the respective degree intervals relative to the total number of nodes in the networks. Numbers at the bottom of the bars are node counts for the R = resting state, S = speech production, and Syl = syllable production networks in the corresponding intervals. The 3-D networks were visualized with the BrainNet Viewer (http://www.nitrc.org/projects/bnv/). Abbreviations: 1 = area 1; 3a/3b = areas 3a/3b; 44 = area 44; 45 = area 45; 4a/4p = anterior/posterior part of area 4; 5Ci = area 5Ci; 5M = area 5M; 6 = area 6; 7A/7P/7PC = area 7A/7P/7PC; 17 = area 17; 18 = area 18; AmCM = subdivision CM of the amygdala; Cbl-V/VIv/IX/IXv = cerebellar lobules V/VIv/IX/IXv; Cd = caudate nucleus; Cu = cuneus; FG = fusiform gyrus; Gpe = external segment of globus pallidus; hIP1-3 = areas hIP1-3; HippFD/SUB = hippocampal subdivisions FD/SUB; hOC4v/hOC5v = ventral parts of areas hOC4/hOC5; IL = insula lobe; IOG/MOG/SOG = inferior/middle/superior occipital gyrus; LG = lingual gyrus; MCC/PCC = middle/posterior cingulate cortex; mFG = medial frontal gyrus; MFG = middle frontal gyrus; MTG = middle temporal gyrus; OP1-4 = operculum; PF/PFm/PFop/PFt/PGa/PGp = areas PF/PFm/PFop/PFt/PGa/PGp in the inferior parietal cortex; Put = putamen; SFG = superior frontal gyrus; TE1.1–3.0 = areas TE1.1–3.0; THp = parietal part of the thalamus. All connectivity matrices are publicly available at http://figshare.com/articles/The_Functional_Connectome_of_Speech_Control/1431873; the codes used to transform the fMRI data to networks can be found at http://research.mssm.edu/simonyanlab/analytical-tools/.

Within the top 30% of most interconnected network nodes in the SPN and RSN (normalized *k*_*i*_ ≥ 0.7; total of 109 nodes), six high-degree hubs were shared, including the premotor cortex (bilateral area 6), primary motor cortex (area 4a), left parietal cortex (area 5M), and right thalamus (Fig 3I–3II, Table 1). All these hubs were among the top 10% of high-degree nodes for both the SPN and RSN (normalized *k*_*i*_ = 0.9–1.0) and did not differ in their degree values when comparing between the SPN and RSN (all *p* ≥ 0.9) (Fig 3IB and 3IC, 3IIB and 3IIC).

Considering the topological structure of connections, all hubs in the RSN exhibited densely connected nodes (min–max = 126–137 connections) but had low link weights (min–max = 57.5–68.4), resulting in a high nodal degree, *k*_*i*,_ but comparatively low nodal strength, *s*_*i*_ (Fig 1III). In contrast, while the SPN hubs showed nodal degree (min–max = 128–132 connections) similar to that of the RSN, they were characterized by higher link weights (min–max = 84.3–89.0) compared to the RSN. Thus, although the number of connections established by these hubs was somewhat similar during both the resting state and speech production, the weights of connected links were higher during speaking.

*Distinct network nodes*. Normalized strength values of the majority of RSN nodes were found in the range 0.4–0.69 (total of 61 nodes), whereas normalized strength values of most SPN nodes were in the higher range of 0.7–0.89 (total of 67 nodes) (Fig 2IV). On the other hand, both the SPN and RSN had their maximum high-degree nodes located within the range 0.7–0.89 (normalized *k*_*i*_), with 67 nodes for SPN and 77 nodes for RSN (Fig 3IV). The shift in nodal strength from medium in the RSN to higher in the SPN reflected their greater influence and the association of these nodes within the SPN community, thus underlying the organizational differences in network topology between the resting state and speaking.

**Fig 4:**
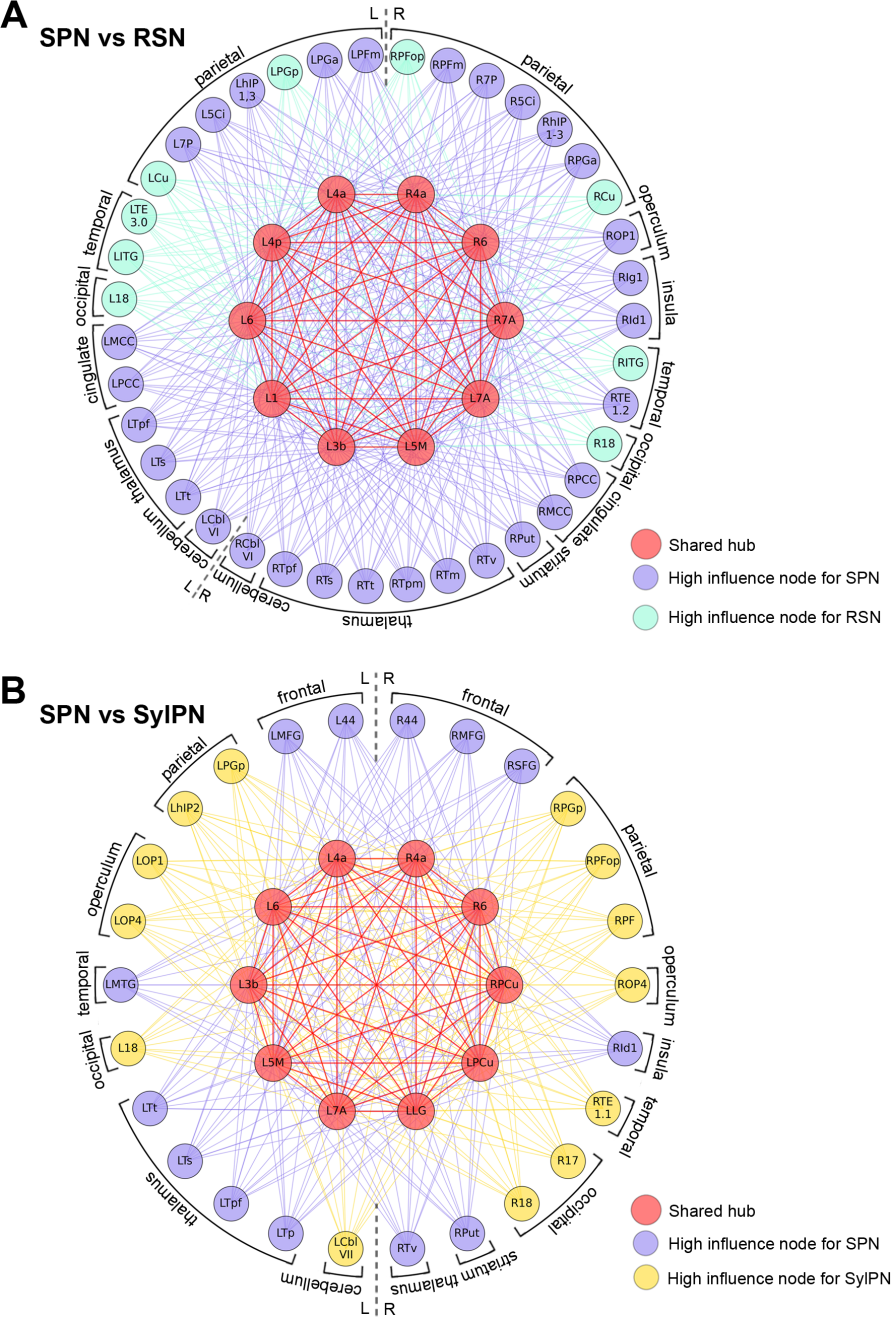
Network formation around shared hubs and high-strength nodes of the group-averaged networks at the minimal density of 60% in RSN versus SPN (A) and SPN versus SylPN (B). Overlap and difference in recruitment of local network communities during rest and sentence production. Red–shared hubs and their connections between the networks; purple–high-strength nodes and their connections in SPN; green–high-strength nodes and their connections in RSN; yellow–high-strength hubs and their connections in SylPN. Abbreviations: L/R = left/right; 3b = areas 3a/3b; 44 = area 44; 4a/4p = anterior/posterior part of area 4; 5Ci = area 5Ci; 5M = area 5M; 6 = area 6; 7A/7P/7PC = area 7A/7P/7PC; 17 = area 17; 18 = area 18; Cbl-VI = cerebellar lobule VI; Cu = cuneus; hIP1-3 = areas hIP1-3; Id1/Ig1 = insular areas Id1 and Ig1; LG = lingual gyrus; MCC/PCC = middle/posterior cingulate cortex; MFG = middle frontal gyrus; MTG = middle temporal gyrus; OP1-4 = operculum; PF/PFm/PFop/PFt/PGa/PGp = areas PF/PFm/PFop/PFt/PGa/PGp in the inferior parietal lobule; Put = putamen; SFG = superior frontal gyrus; TE1.1–3.0 = areas TE1.1–3.0; Tp/pf/s/t/m/pm/v = parietal/prefrontal/somatosensory/temporal/motor/premotor/visual divisions of the thalamus. All connectivity matrices are publicly available at http://figshare.com/articles/The_Functional_Connectome_of_Speech_Control/1431873; the codes used to transform the fMRI data to networks can be found at http://research.mssm.edu/simonyanlab/analytical-tools/.

Specifically, the SPN but not the RSN showed significantly higher-strength nodes (normalized *s*_*i*_ = 0.7–1.0) in the bilateral inferior parietal lobule, including the supramarginal (area PFm) and angular (area PGa) gyri, the intraparietal sulcus (areas hIP1-3), parietal cortex (areas 5Ci, 7P), middle/posterior cingulate cortex, thalamus, and cerebellum (lobule VI), as well as the right auditory cortex (area TE1.2), insula (areas Ig1, Id1), operculum (OP1), and putamen (all corrected *p* ≤ 0.048) (Fig 2IIB and 2IIC, Fig 4A, Table 1). SPN-specific high-degree nodes (normalized *k*_*i*_ = 0.7–1.0), which were not present in the RSN within the same range, included the right primary somatosensory cortex (area 1), operculum (area OP1), insula (area Id1), intraparietal sulcus (areas hIP1-3), auditory cortex (areas TE1.1–1.2), middle temporal gyrus, putamen, globus pallidus, left posterior cingulate cortex, and bilateral thalamus (Fig 3IIB and 3IIC, Table 1). However, the degree values of these nodes did not differ between the SPN and RSN (all *p* > 0.05) due, in part, to identical connection densities in both networks.

Conversely, the RSN but not the SPN had high-strength nodes (normalized *s*_*i*_ = 0.7–1.0) in the bilateral inferior parietal lobule (left angular (area PGp) and right supramarginal (area PFop) gyri), inferior temporal gyrus, cuneus, occipital cortex (area 18), and the left auditory cortex (area TE3.0) (Fig 2IB and 2IC, Fig 4A, Table 1). In addition, the RSN but not the SPN had high-degree nodes in the bilateral inferior temporal gyrus, occipital cortex (left area 18 and right areas 17 and 18), operculum (area OP4), right inferior frontal gyrus (area 45), and left cuneus (Fig 3IB and 3IC, Table 1). These nodes did not show significant differences in either strength or degree values between the RSN and SPN (all *p* ≥ 0.41).

#### Nodal attributes of SPN and SylPN

**Fig 5:**
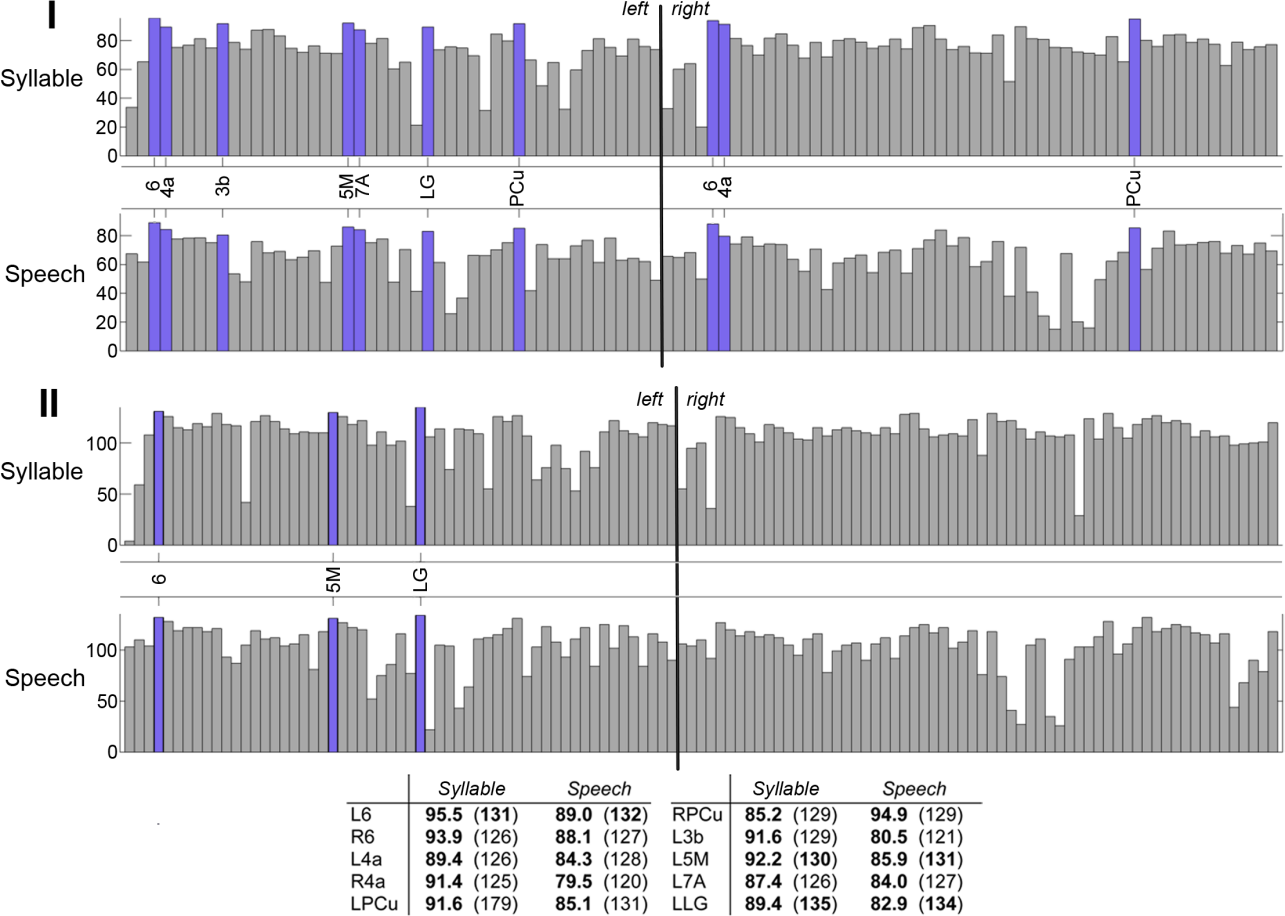
Shared high-degree and high-strength hubs in the group-averaged SPN and SylPN at the minimum density of 60%. (I) Bar charts show strength values of the top 30% strongest nodes (normalized *s*_*i*_ ranges 1.0–0.9 and 0.89–0.7 in Fig 4) in both the SPN and SylPN. Blue bars highlight nodes that are strength hubs in both SPN and SylPN. (II) Bar charts of the same format show shared-degree hubs of the SPN and SylPN among the top 30% most interconnected nodes (normalized *k*_*i*_ ranges 1.0–0.9 and 0.89–0.7 in Fig 5). The table shows nodal values of strength and degree in parentheses. Bold numbers indicate that a node is a hub with respect to the respective metric. Abbreviations: 3b = area 3b; 4a = area 4a; 5M = area 5M; 6 = area 6; 7A = area 7A; LG = lingual gyrus; PCu = precuneus. The corresponding data are publicly available at http://figshare.com/articles/The_Functional_Connectome_of_Speech_Control/1431873.

*Shared network hubs*. Similar to the SPN and in contrast to the RSN, the SylPN showed high values of nodal strength and degree. Among the top 30% of strongest network nodes (normalized *s*_*i*_ ≥ 0.7, total of 101 nodes), the SylPN and SPN shared ten high-strength hubs, which were located in the premotor (bilateral area 6), primary motor (bilateral area 4a), primary somatosensory (left area 3b) and parietal cortices (left areas 5M and 7A), the left lingual gyrus and bilateral precuneus (Fig 5I, Table 2). All these hubs were within the top 10% of network strength for both the SylPN and SPN (normalized *s*_*i*_ = 0.9–1.0), except for the right primary motor cortical hub in the SPN, which was located in the top 20%–30% of network strength (normalized *s*_*i*_ = 0.7–0.89) (Fig 2IIB and 2IIC, 2IIIB and 2IIIC).

**Table 2:** Top 30% shared and distinct hubs and high-influence nodes in the SylPN and SPN (normalized strength/degree range of 0.7–1.0).

Shared hubs in SylPN and SPN	Distinct nodes in SPN but not SylPN	Distinct nodes in SylPN but not SPN
*Strength*		
Premotor cortex	Inferior frontal gyrus	Operculum
L Area 6 ‒ SylPN 95.5; SPN 89.0	L Area 44 ‒ 61.6	L Area OP1, OP4–76.3 ± 3.2
R Area 6 ‒ SylPN 93.8; SPN 88.1	R Area 44–68.2	R Area OP4–67.9
Primary motor cortex	Middle frontal gyrus	Inferior parietal lobule
L Area 4a ‒ SylPN 89.4; SPN 84.3	L– 67.4	L Angular gyrus (area PGp)– 71.2
R Area 4a ‒ SylPN 91.4; SPN 79.5	R– 64.9	R Angular gyrus (area PGp)– 74.1
	Superior frontal gyrus	R Supramarginal gyrus (areas PF, PFop)– 77.4 ± 3.9
	R– 65.7	
Primary somatosensory cortex	Middle temporal gyrus	Intraparietal sulcus
L Area 3b ‒ SylPN 91.6; SPN 80.5	L– 70.4	L Area hIP2–81.2
Parietal cortex	Thalamus	Auditory cortex
L Area 7A ‒ SylPN 87.4; SPN 84.0	L Prefrontal, Parietal, Somatosensory, Temporal subdivision– 68.7 ± 5.4	R Area TE1.1 ‒ 71.5
L Area 5M ‒ SylPN 92.1; SPN 85.9		
	R Visual subdivision– 67.8	
Lingual gyrus	Putamen	Occipital cortex
L ‒ SylPN 89.4; SPN 82.9	R– 62.0	L Area 18–66.5
		R Areas 17 and 18–73.8 ± 5.6
Precuneus	Insula	Cerebellum
L ‒ SylPN 91.6; SPN 85.1	R Area Id1–61.9	L Lobule VII– 73.8
R ‒ SylPN 94.9; SPN 85.2		
*Degree*		
Premotor cortex	Middle frontal gyrus	Operculum
L Area 6 ‒ SylPN 131; SPN 132	L– 110	L Area OP4 ‒ 117
	Superior frontal gyrus	
	L– 103	
	R ‒ 107	
Parietal cortex	Insula	Inferior parietal lobule
L Area 5M ‒ SylPN 130; SPN 131	R Area Ig1 ‒ 105	L Angular gyrus (area PGp)– 110
		R Angular gyrus (area PGp)– 109
		R Supramarginal gyrus (areas PF,
		PFop) ‒ 110
Lingual gyrus	Thalamus	Auditory cortex
L ‒ SylPN 135; SPN 134	L Motor, premotor, somatosensory subdivisions– 102.3 ± 9.0	L Areas TE1.1, 1.2, 3.0–102.3±7.5
	Globus pallidus, external segment	Occipital cortex
	R ‒ 93	L Area 18–106
		R Area 17 ‒ 121
		Cerebellum
		L Lobule V, VII– 111.5 ± 7.8
		R Lobule V, VI– 100.5 ± 0.7

Left column: Shared high-strength and high-degree hubs between syllable production network (SylPN) and speech production (SPN) networks. Middle column: Distinct high-strength and high-degree nodes in SPN but not SylPN. Right column: Distinct high-strength and high-degree nodes in SylPN but not SPN. Values indicate the strength or degree of the node, respectively. Mean ± SD is given for those nodes that are in similar cytoarchitectonic regions. R–right; L–left.

Within the top 30% of connected network nodes (normalized *k*_*i*_ ≥ 0.7, total of 119 nodes), three high-degree hubs were shared by the SylPN and SPN, including the left premotor cortex (area 6), parietal cortex (area 5M), and lingual gyrus (Fig 5II, Table 2). All hubs for both the SPN and SylPN were within the range of normalized *k*_*i*_ = 0.9–1.0 (Fig 3IIB and 3IIIB). The degree and strength of hubs was similar between the SPN and SylPN (all *p* ≥ 0.05).

*Distinct network nodes*. The SPN but not the SylPN had high-strength nodes (normalized *s*_*i*_ = 0.7–1.0) in the bilateral inferior frontal (area 44), middle and superior frontal gyri, left middle temporal gyrus, bilateral thalamus, right putamen, and insula (area Id1), whereas SPN-specific high-degree nodes were found in the bilateral superior and left middle frontal gyri, right insula (area Ig1), left thalamus, and right globus pallidus (Fig 2IIB and 2IIC, 2IIIB and 2IIIC, Fig 4B, Table 2). On the other hand, the SylPN but not the SPN showed high-strength and high-degree nodes in the bilateral operculum (areas OP1 and OP4), bilateral angular gyrus (area PGp), right supramarginal gyrus (areas PF and PFop), left intraparietal sulcus (area hIP2), right auditory cortex (area TE1.1), bilateral occipital cortex (areas 17 and 18), and cerebellum (lobule VII) (Fig 4B, Table 2). Strength and degree of high-strength and high-degree nodes were similar in the SPN and SylPN (all corrected *p* > 0.05).

### Experiment 2: Community-Based Architecture of Functional Networks during the Resting State, Speech-Related and Non-speech-related Tasks

To identify the global configuration and characteristic features of the SPN, we performed a community-based analysis of functional networks during the RSN, SylPN, and SPN, as well as during the performance of an unrelated motor task of sequential finger tapping (finger-tapping network; FTN) and an unrelated auditory task of pure tone temporal discrimination (auditory discrimination network; ADN). To estimate network communication patterns, we classified high-strength hubs into connector (linking communities) and provincial hubs (connecting nodes within the same module) based on their participation in intermodular versus intramodular links [58].

**Fig 6:**
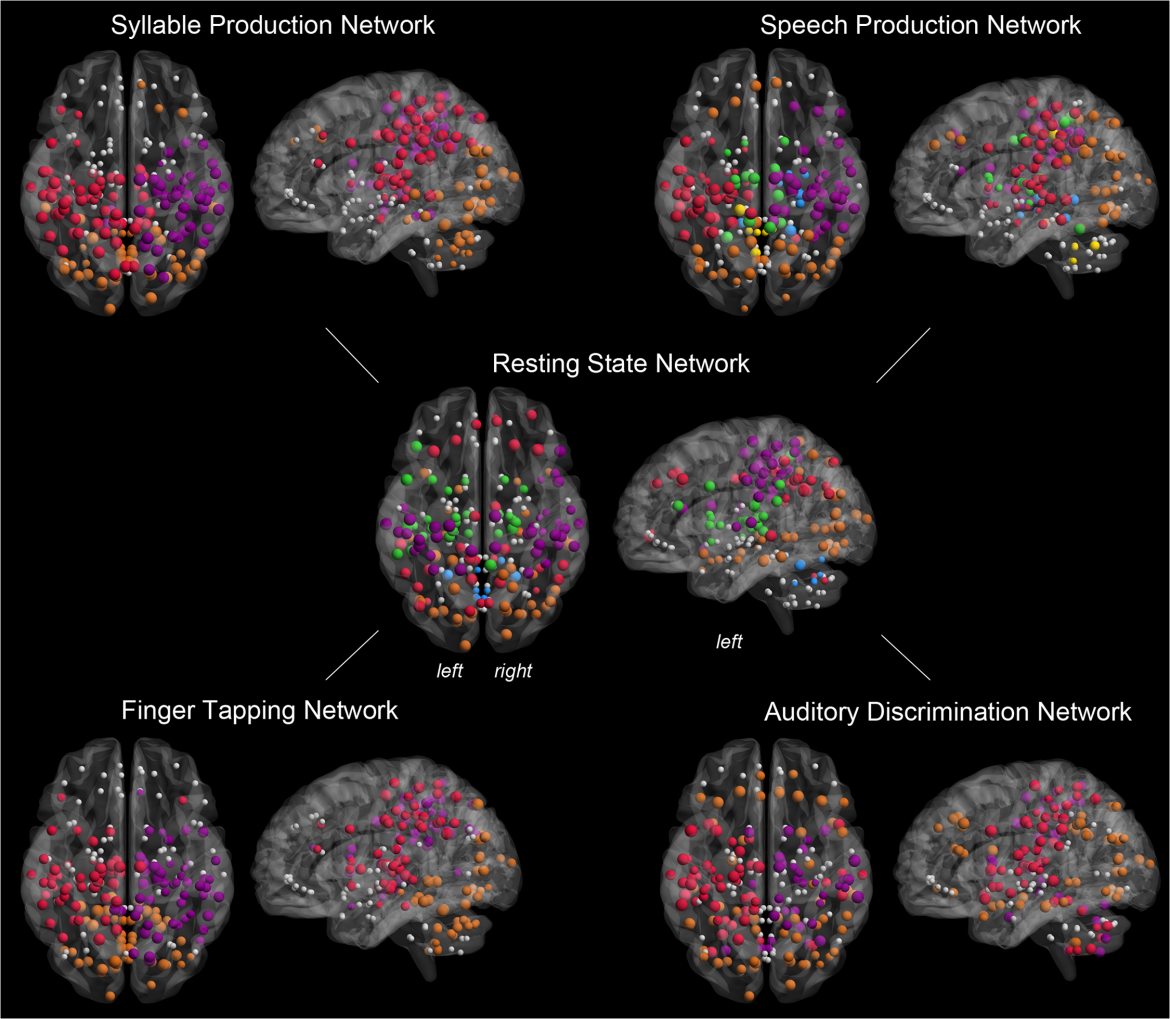
Modular topology of the group-averaged networks during the resting state, syllable production, sentence production, sequential finger tapping, and auditory discrimination of pure tones. Spatially distributed network communities are shown on 3-D brain renderings in the axial and sagittal views and are color-coded based on nodal module affiliation. Nodal size indicates normalized degree; nodes that were removed by the employed elimination strategy are shown in gray. All connectivity matrices are publicly available at http://figshare.com/articles/The_Functional_Connectome_of_Speech_Control/1431873; the codes used to transform the fMRI data to networks can be found at http://research.mssm.edu/simonyanlab/analytical-tools/.

**Fig 7:**
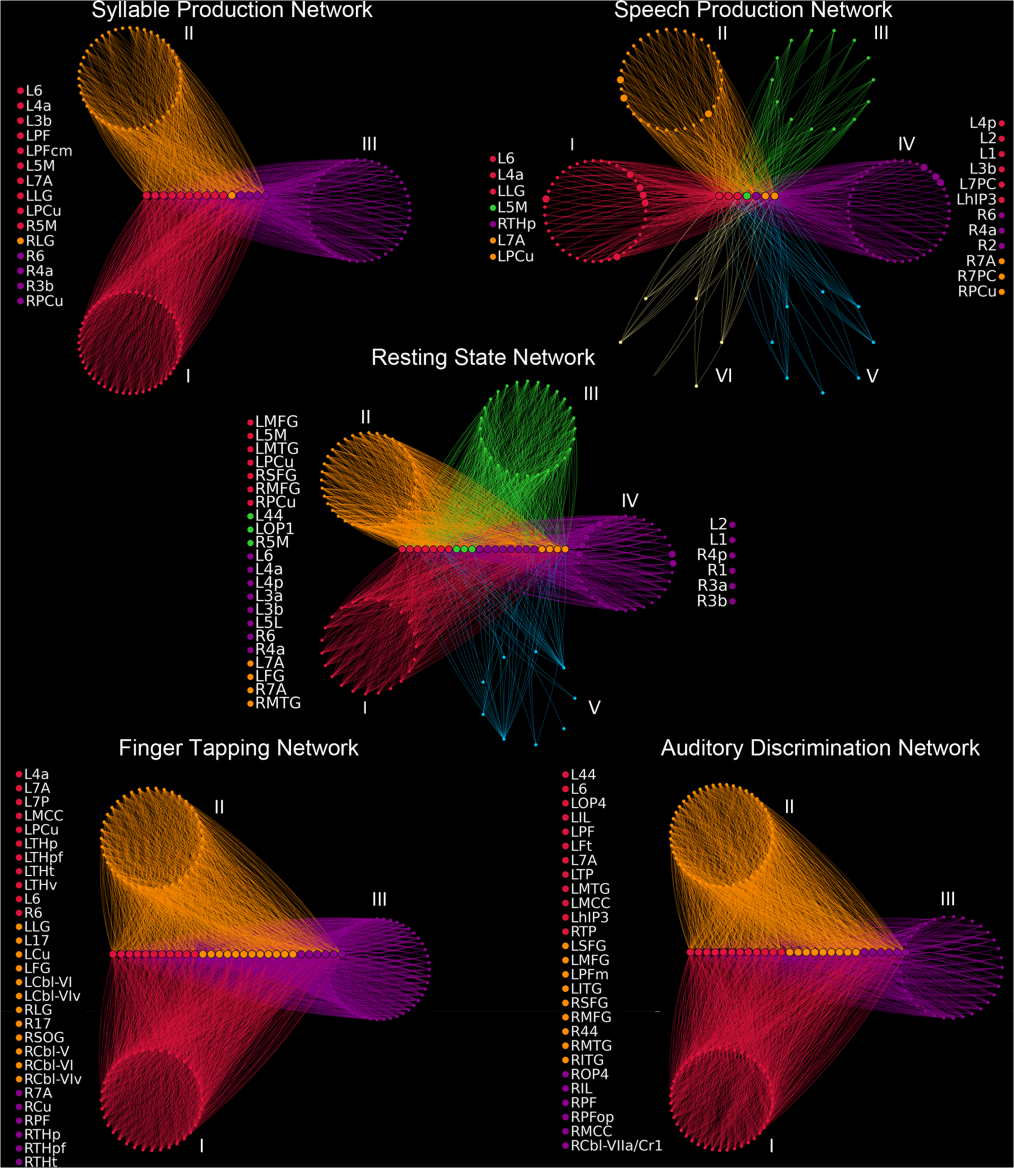
Functional community structure of the group-averaged networks during the resting state, syllable production, sentence production, sequential finger tapping, and auditory discrimination of pure tones. Network modules are shown as circular groups of nodes positioned around the respective connector hubs, which are arranged on horizontal lines. Only edges passing through connector hubs are drawn, with the respective edge colors indicating the community affiliation of the target nodes. Provincial hubs are displayed as larger circles within their respective native communities. Nodal colors illustrate module membership, where colors are matched to Fig 6. Node lists on the left and right of each graph specify connector and provincial hubs, respectively. Abbreviations: 1 = area 1; 17 = area 17; 2 = area 2; 3a/3b = areas 3a/3b; 44 = area 44; 4a/4p = anterior/posterior part of area 4; 5L/5M = area 5L/5M; 6 = area 6; 7A/7P/7PC = area 7A/7P/7PC; Cbl-V/VI/VIv/VIIa/Cr1 = cerebellar lobules V/VI/VIv/VIIa/Cr1; Cu = cuneus; FG = fusiform gyrus; hIP3 = areas hIP3; IL = insula; SOG = superior occipital gyrus; ITG/MTG = inferior/middle temporal gyrus; LG = lingual gyrus; MCC = middle cingulate cortex; OP1-4 = operculum; PCu = precuneus; PF/PFm/PFop/PFt/PGa/PGp = areas PF/PFm/PFop/PFt/PGa/PGp in the inferior parietal cortex; MFG = middle frontal gyrus; THp/THpf/THpm/THt = parietal/prefrontal/premotor/temporal part of the thalamus; TP = temporal pole; R–right; L–left. All connectivity matrices are publicly available at http://figshare.com/articles/The_Functional_Connectome_of_Speech_Control/1431873; the codes used to transform the fMRI data to networks can be found at http://research.mssm.edu/simonyanlab/analytical-tools/.

All functional networks exhibited different patterns of modularity; while the RSN consisted of five distinct modules, the SylPN, FTN, and ADN scaled down to three modules, and the SPN expanded to six modules (Fig 6). During the resting state, functional network communities spanned (I) the frontoparietal cortex, (II) the temporooccipital cortex, (III) the insula/opercular cortex, basal ganglia, and thalamus, (IV) the sensorimotor and parietal cortex, and (V) the cerebellum. The RSN was characterized by a large number of connector hubs compared to relatively few provincial hubs (22 versus 6 hubs, respectively). Connector hubs linking different communities originated from modules I–IV and included sensorimotor and parietal cortical regions, whereas provincial hubs were centered on the primary sensorimotor cortex and exclusively recruited from module IV (Fig 7).

Task performance dramatically changed the RSN modular configuration. The nodal community structure of the SylPN consisted of three modules and was characterized by two large communities (modules I and III), which were located within the left and right hemispheres (Fig 6), respectively, comprising 70% of all network nodes (105 out of a total 150 nodes, Fig 7). These relatively symmetrical modules predominantly included the sensorimotor, inferior frontal, inferior parietal, and superior temporal cortical regions, the basal ganglia, and the thalamus, whereas a smaller module II included mainly cerebellar and occipital regions. Hub classification revealed that all SylPN hubs participated as connector hubs and were situated around the sensorimotor and inferior parietal cortical regions with the left-sided module I containing the majority of connectors (10 out of 15 hubs).

Speech production introduced the most complex network community architecture with the emergence of six distinct modules, which represented the largest number of modules compared across all experimental conditions. While left- and right-hemispheric modules I and IV of the SPN included predominantly sensorimotor, inferior frontal, inferior parietal, insular/opercular, and temporal cortical regions and largely corresponded to modules I and III of the SylPN, the new and extended nodal communities were formed and centered around the frontoparietooccipital cortex (module II), the basal ganglia and thalamus (module III), the hippocampus and thalamus (module V), and the cerebellum (module VI). The SPN was further characterized by a low ratio of connector to provincial hubs (7 versus 12 hubs, respectively), suggesting a high degree of network segregation with only a few network-wide coordinators.

Similar to the SylPN, nonlinguistic ADN and nonspeech motor FTN were comprised of three network communities of a distinct spatial profile (Fig 7). The FTN showed a community structure comparable to SylPN with symmetrical modules I and III located within the left and right hemispheres (Fig 6), respectively, and connector-only hubs linking the three nodal communities (Fig 7). This finding points to a lack of locally controlled information flow within nodal communities in both the FTN and SylPN, which is reflective of the low complexity of these two motor production tasks. In contrast to the SylPN and FTN, which largely lacked the involvement of the frontal cortical regions, the ADN formed an extensive module II, which included the vast majority of frontal and parietal regions, bilaterally. Compared to the ADN, the SPN recruited a smaller subset of the frontoparietal regions, which were distributed across five different modules (I–IV and VI), suggesting intramodular importance of particular frontoparietal regions within the SPN.

To quantify the observed differences in network community divisions across all experimental conditions, we assessed the partition distance *p*_*d*_ between network community structures by calculating normalized mutual information coefficients between the respective partition vectors across all networks. Comparing the SPN to all other conditions, the highest degree of similarity with respect to their partitions was found with the SylPN (*p*_*d*_(SPN,SylPN) = 0.27), followed by the RSN (*p*_*d*_(SPN,RSN) = 0.25). The FTN community structure exhibited less correspondence to the SPN (*p*_*d*_(SPN,FTN) = 0.23), whereas the ADN showed the most discordance in its modular architecture compared to the SPN (*p*_*d*_(SPN,ADN) = 0.15), confirming the distinctly different topologies of the non-speech-related networks.

Finally, based on our finding that primary sensorimotor and premotor cortices contributed to the formation of a shared hub network across all experimental conditions (Figs 4, 6, and 7), we examined the existence of adaptive flexible hubs in these sensorimotor regions by comparing their participation coefficients, *p*_*ci*,_ estimating the uniformity of connections of these regions across all networks [59,60]. Compared to the frontoparietal regions, which were recently reported to host flexible hubs rapidly adapting their connectivity patterns depending on task demands [60], we found that the shared sensorimotor hubs in areas 6, 4a, 4p, 3b, and 1 across the RSN, SylPN, SPN, FTN, and ADN also show high participation coefficients similar to shared frontoparietal hubs (sensorimotor versus frontoparietal *p*_*ci*_: 0.62 ± 0.15 versus 0.67 ± 0.07, *p* = 0.11).

## Discussion

Guided by the concept of network integration, segregation, and influence, we investigated the organization of the functional speech connectome and characterized the evolution of its local and global network attributes from the resting state to syllable production as a speech-relevant motor task with minimal linguistic meaning to complex production of meaningful sentences as a real-life speech behavior in comparison with the production of the nonlinguistic task of pure tone discrimination and the nonrelevant motor task of sequential finger tapping.

Among all experimental conditions, the SPN showed the most complex architecture with the highest degree of network segregation. Compared to the RSN, the closely related SPN and SylPN exhibited pronounced shifts to stronger inter-regional correlations, higher global efficiency (i.e., short path length), and higher clustering coefficient (i.e., the cliquishness of connections) that are characteristic of a small-world network [61,62]. These findings point to increases in neural communication and processing within the SPN and SylPN that are achieved by utilizing a more cost-efficient information transfer across highly engaged local neural communities in order to meet the demands of increased task complexity from rest to syllable production to speaking.

Compared to all examined networks, the SPN was further characterized by several striking topological features contributed by (i) the regions constituting a hub network; (ii) the connectivity profile of hub networks, and (iii) community-based network organization, highlighting the formation of sensorimotor flexible hubs.

### The SPN Hub Network

The formation of hubs and their roles within and across network communities provided detailed insights into the functional specialization of the speech connectome (Figs 4 and 7). We found that the SPN shared common hubs with the RSN and SylPN, constituting a core hub network, which was centered on the left laryngeal and orofacial regions of the primary motor cortex and its main input regions in the surrounding premotor, somatosensory, and parietal cortices. Notably, the strength of SPN hubs was significantly greater than those of the RSN or SylPN, which may be explained by the task complexity. Our data further highlighted the role of the left area 4p as an important core hub during speaking. The area 4p (posterior part of the primary motor cortex) is known to be involved in initiation and execution of motor commands and the modulation of movement-related attention. A recent meta-analysis of speech-related fMRI literature showed that the peak of activity within the laryngeal motor cortex is located in the area 4p [63]. Conversely, the area 4a (anterior part of the primary motor cortex) functionally resembles the secondary motor cortex and requires higher order sensory feedback for motor execution [64–67]. Our findings demonstrate that the left area 4p but not either left or right area 4a has distinctive strength within the core hub network during speaking compared to syllable production and the resting state. The importance of area 4p within the SPN is further emphasized by its role as a provincial hub, which established dense intramodular connections and largely influenced its own nodal community comprised of the majority of sensorimotor core hub regions contributing to the final common cortical pathway of the SPN. In contrast, area 4a served as a connector hub, fulfilling its role in network-wide information integration not limited to a particular module. Based on the distinct involvement of area 4p and area 4a, these two primary motor cortical subdivisions may differentially influence the hub network connectivity for formation of long-range neural connections during speech production compared to other conditions.

### The Connectivity Profile of the SPN Hub Network

Major differences between the SPN and all other networks were found in their hub network connectivity patterns. Detailed investigation of the connectivity profiles of the SPN compared to the RSN and SylPN showed that, while these networks share the same sensorimotor hubs, the connections of the common hub network with other brain regions largely varied depending on the state of brain activity (i.e., resting, syllable production, or speaking) (Figs 4 and 7). The same finding held true when comparing the architecture of the SPN with the FTN and ADN.

Being centrally embedded within the network, the sensorimotor hubs recruited an exclusive set of high-degree and high-strength nodes in the SPN. Compared to the RSN, the SPN-only brain regions included the parietal operculum, insula, middle/posterior cingulate cortex, putamen, thalamus, and cerebellum, while the parietal and temporal cortex had different regions specifically designated to either the SPN or RSN (Fig 4A). Comparisons between the SylPN and SPN showed that the SPN recruited the prefrontal cortex, insula, putamen, and thalamus, whereas the SylPN involved the parietal, temporal, and occipital cortices, and the cerebellum (Fig 4B). These data demonstrate that the transition from the resting state to meaningless syllable production to meaningful speaking is contingent upon the involvement of the multimodal associative regions, such as the frontoparietal cortices as well as the basal ganglia and cerebellum (see Discussion in S1 Text for the discussion of subcortical structures).

In particular, compared to the resting state, the SPN recruited a wide array of brain regions across multiple nodal communities (Fig 6). The SPN core sensorimotor hub network established connections with the brain regions responsible for sound perception and encoding (auditory cortex), phonological and semantic processing (parietal cortex), lexical decisions and narrative comprehension (middle/posterior cingulate cortex), motor planning and temporal processing of auditory stimuli (insula), control of learned voice production and suppression of unintended responses (basal ganglia), and modulation of vocal motor timing and sequence processing (cerebellum) (reviewed in [1,68]). In contrast to the SylPN, the SPN further refined its network properties and functional specializations by recruiting brain regions responsible for high-order planning and processing. Among these regions, the involvement of the prefrontal cortex was particularly prominent, as a strong relationship with the core hub network was found only in the SPN but not in the SylPN (Fig 2II–2IIIC). Similar to the SylPN, the speech-unrelated FTN also showed no prefrontal component within its large-scale network, which attests to the similarities between the simple vocal motor task of syllable production and the simple motor task of finger tapping, as well as further underscores the distinct prefrontal involvement in the SPN. However, the question arises as to whether the prefrontal cortical participation is related more to general cognitive processing than speech production. Comparisons between the SPN and the nonlinguistic decision-making ADN showed that while both networks shared common nodes in the prefrontal cortex, the SPN required only a specialized subset of the prefrontal region, which was additionally assigned to different neural communities as opposed to forming one large frontoparietal community in the ADN. Such network topology suggests that the prefrontal cortex may play a specialized role in the formation of cognitive aspects of speech control, such as verbal fluency, semantic context associations and violations, word retrieval and sentence generation, stimulus monitoring, and attention-demanding speech comprehension [69–76], which are not characteristic of the production of meaningless syllables, other simple motor or speech-unrelated cognitive tasks. Taken together, our study demonstrates that the production of a syllable, as a speech building block, leads to an integrated network configuration dominated by connector hub regions with almost no participation of the prefrontal cortex in the large-scale network, while the ability to produce and monitor meaningful speech requires locally segregated information processing by specialized communities and information transfer through the prefrontal cortical regions within the SPN.

### Community-Based Network Organization

To emphasize that the changes in network topology observed from resting to syllable production to speaking truly represent characteristics of speech-relevant motor networks, we contrasted the network architecture of the RSN, SPN, and SylPN with the topology of the FTN representing a nonspeech related motor control task and of the ADN as an example of a nonlinguistic decision-making task. Our findings demonstrated once again that among all examined conditions, the SPN showed a unique functional architecture (Figs 6 and 7) that reflected the high level of complexity of meaningful speech as opposed to repetitive syllable production, finger tapping, and auditory temporal discrimination. While the SylPN exhibited a connectivity profile similar to the SPN on a nodal level (Figs 2 and 3), the recruitment of nodes into network communities showed a distinctly more complex and segregated network organization in the SPN than in the SylPN (Fig 6). In contrast, the topology of the SylPN, FTN, and ADN was characterized by the emergence of three nodal communities, which were, however, distinctly configured depending on task demands. The SylPN had two large, relatively symmetrical left and right communities with the left component containing the majority of the connector hubs; the FTN had its connector hubs evenly distributed across both hemispheres, whereas the community partition of the ADN illustrated the distributed functional couplings of frontoparietal areas across the network (orange module in Fig 6).

As all examined networks had commonly shared hubs in the primary sensorimotor and premotor cortical regions, it is plausible to suggest that behaviorally driven organization and functional specialization of the core sensorimotor network (hubs) defined the unique topological arrangement of large-scale networks, especially in the SPN and SylPN. Based on the participation of the same sensorimotor hubs in several functional domains across different networks and their ability to adaptively establish connectivity with a large range of brain regions (Fig 7), the detected sensorimotor hubs may be considered as flexible network hubs [60,77] with a potential capacity for operational heterogeneity. This novel finding challenges the previous concept of the sensorimotor cortex to exert only low order unimodal influences on other regions [77–80].

### Conclusion

In summary, several new findings emerged from our utilization of multivariate graph theoretical analysis of the SPN. Specifically, combining the analysis of functional interactions at the level of network communities with the assessment of individual nodes provided detailed quantitative evidence suggesting that speech production requires specialized network organization around the core local communities centered on the sensorimotor cortex. Because of their high strength, degree, and heterogeneity of functional connections and participation across various behaviors, these sensorimotor regions may be considered to be multimodal flexible network hubs. Among these, area 4p of the primary motor cortex emerged as a particularly important cortical hub in speech controlling network. Furthermore, the production of real-life speech depended on the proper orchestration of a large-scale network, comprised of specialized cortical and subcortical nodes in the prefrontal cortex, insula, putamen, and thalamus, which were less important for other networks, including the SylPN. Collectively, these individual nodes and their roles within functionally specialized nodal communities determined the reconfiguration of global network architecture from the resting state to syllable production to speaking and clarified the distinct functional specializations of core sensorimotor hub regions within large-scale neural networks.

## Materials and Methods

### Ethics Statement

Written informed consent was obtained from all subjects prior to study participation, which was approved by the Institutional Review Boards of the Icahn School of Medicine at Mount Sinai and National Institute of Neurological Disorders and Stroke, National Institutes of Health.

### Subjects

Twenty-seven right-handed monolingual English-speaking healthy subjects (18 females, 9 males, age 52.2 ± 11.3 years [mean ± SD]) without any history of neurological, psychiatric, or laryngeal disorders participated in the study. Among these, 20 subjects (13 females, 7 males, age 55.2 ± 9.8 years) participated in the initial resting state and speech production fMRI study. As a follow-up study, 14 subjects (5 same and 9 new; total: 7 females, 7 males, age 52.0 ± 13.1 years) participated in the other task-production fMRI studies, including syllable production, sequential finger tapping, and pure tone auditory temporal discrimination. To ensure the compatibility of data from different subject groups, we conducted a comparison of time series in all hub regions in the two groups of subjects from the original and follow-up studies and found no statistically significant differences between these groups (all *p* > 0.07 adjusted for family wise error [FWE] based on the maximal statistic T_max_ [81]), indicating that these data were coherent and not biased by intersubject differences.

Written informed consent was obtained from all subjects prior to study participation, which was approved by the Institutional Review Boards of the Icahn School of Medicine at Mount Sinai and National Institute of Neurological Disorders and Stroke, National Institutes of Health.

### Data Acquisition

Brain images were acquired on a 3.0 Tesla GE scanner equipped with a quadrature birdcage radio frequency head coil (Milwaukee, WI).

#### Resting-state fMRI (rs-fMRI)

Whole brain rs-fMRI images were acquired before the task-production fMRI within the same scanning session using gradient-weighted echo planar imaging (EPI) (150 contiguous volumes with TR 2 s, TE 30 ms, flip angle 90°, 33 slices, in-plane resolution 3.75 mm). The subjects were instructed to rest without specific thoughts, with their eyes closed in an environment with dimmed light. Physiological recordings included measurements of respiration volume and heart rhythm sampled at 50 Hz with the recording onset triggered by the scanner’s selection trigger pulse.

#### Task-production fMRI

Whole brain images during syllable production, sentence production, and the auditory discrimination task were acquired using gradient-weighted EPI pulse sequences (total TR 10.6 s (8.6 s for task + 2 s for image acquisition) during syllable and speech production; total TR 8.9 s (6.9 s for task + 2 s for image acquisition) during auditory discrimination; TE 30 ms, flip angle 90°, in-plane resolution 3.75 mm, 36 slices) with BOLD contrast and a sparse-sampling event-related design. All sample stimuli were acoustically presented one at a time and performed by the subject one per acquisition volume. A total of 36 trials per task and 24 resting fixations as a baseline were acquired over the five scanning sessions in each subject with the tasks pseudorandomized between sessions and participants.

During the syllable production task, subjects were instructed to produce four repetitions of the syllable /iʔi/, which consisted of the vowel /i/ as in the word “beet” or “green” followed by a glottal stop /ʔ/ and then the vowel /i/ again as previously described [7,82] (phonetic spelling of the syllable is according to the International Phonetic Alphabet). The syllable /iʔi/ was chosen to achieve maximal vocal fold adduction necessary for voice production. While the syllable /iʔi/ is used during speech production, it is largely devoid of semantic meaning when used as an isolated syllable production task in monolingual native English speakers. During the speech production task, subjects were instructed to produce ten different meaningful, grammatically correct English sentences (e.g., “We are always away”, “Tom is in the army”), one at a time. Different sentences were used to exploit different phonological and lexical elements present in normal speech, while minimizing working memory build-up following the repetition of one sentence during the entire scanning session. Conversely, the choice of the simple and meaningless syllable /iʔi/ was made to achieve task homogeneity, restrict cognitive and linguistic processing, and “focus” brain activity on simple vocal motor control associated with the production of a speech sound. In broader terms, we aimed to use syllable production for narrowing down on speech motor output, while expanding on higher-order processing during the production of different sentences. Because our previous study showed that the production of different syllable types recruits nearly identical functional networks [7], the lack of variation in syllable responses as opposed to the variation in sentence stimuli and responses was not of a concern from the point of functional network organization.

The auditory temporal discrimination task included three pairs of low pure tones at 950 Hz and of 400 ms in duration, which were presented either simultaneously with no interstimulus interval between the two tones or subsequently with a large interstimulus interval of 440 ms from the start of the first stimulus or a short interstimulus interval of an average 39 ms (range 10–80 ms) from the start of the first stimulus. The short interstimulus interval was individually established in each subject during offline testing prior to the scanning session and represented each subject’s individual auditory temporal discrimination threshold, that is the shortest time at which the subject perceived the two stimuli as being asynchronous. All tones were generated at 14,440 Hz sampling frequency and normalized to have the same root mean square (RMS) amplitude as described earlier [83,84]. Subjects listened to the stimuli, one pair at a time, and decided whether a pair of tones was “same” (i.e., simultaneously presented) or “different” (i.e., subsequently presented).

Whole brain images during the finger-tapping task were acquired using gradient-weighted EPI pulse sequences (TR 2 s; TE 30 ms, flip angle 90°, in-plane resolution 3.75 mm, 36 slices) with BOLD contrast and block design. The subjects were visually cued by a picture of a hand to perform sequential finger tapping (i.e., 1-2-3-4-5-4-3-2-1) on a fiber-optic button response unit (Celeritas; Psychology Software Tools) using the dominant right hand. All subjects performed finger tapping for a period of 30 s followed by a 30 s resting period, during which the subjects fixated at a cross on the screen in front of their eyes. Subjects performed a total of five blocks of sequential finger tapping alternated with five blocks of resting fixation.

#### High-resolution MRI

A high-resolution T1-weighted image was collected for anatomical reference using 3-D magnetization-prepared rapid acquisition gradient echo (MPRAGE: TI 450 ms, TE 3.0 ms, flip angle 10°, 128 slices, slice thickness 1.2 mm).

### Data Pre-processing

Data preprocessing was performed using AFNI software [85] following standard steps.

#### rs-fMRI

After the removal of the first four volumes and slice time correction, the rs-fMRI time series were aligned to the high-resolution anatomical volume using a rigid-body transformation. The hardware-related noise in the time series was regressed out based on the anatomy-based correlation corrections (ANATICOR) model [86]; the physiological noise was regressed out based on the retrospective image correction (RETROICOR) model [87]. The global signal was not regressed from rs-fMRI data in order to avoid spurious negative correlation values and negatively correlated networks [88–90]. Residual time series were spatially smoothed with a 6 mm Gaussian kernel within the gray matter and normalized to the standard AFNI space of Talairach-Tournoux.

#### Task-production fMRI

After discarding the first two volumes, all EPI datasets were registered to the volume collected closest in time to the high-resolution anatomical scan using heptic polynomial interpolation, spatially smoothed with a 6-mm Gaussian filter, normalized to the percent signal change and to the standard Talairach-Tournoux space. The task-related responses were analyzed using multiple linear regressions with a single regressor for each task convolved with a canonical hemodynamic response function.

### Network Construction

**Fig 8:**
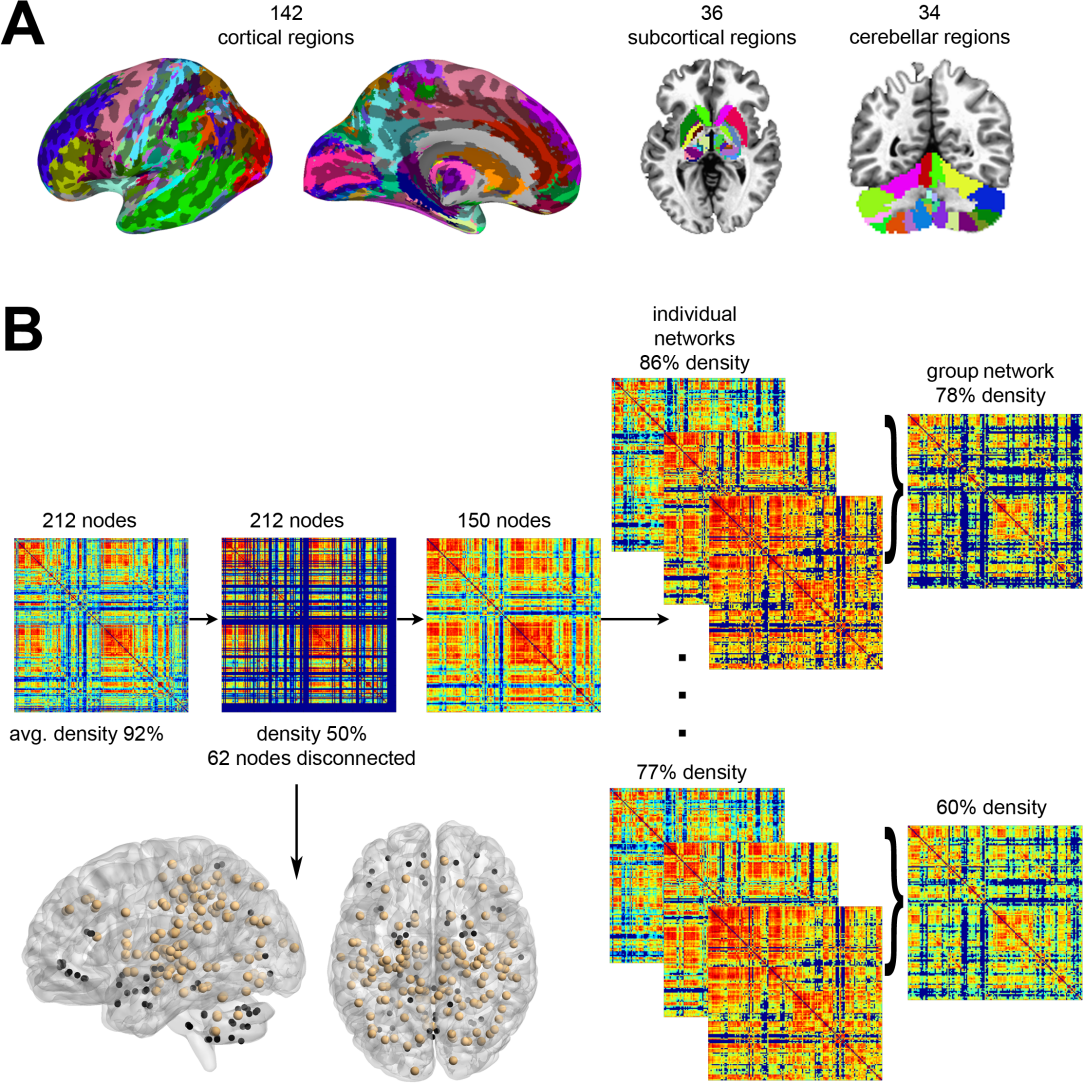
Schematic overview of (A) whole-brain parcellations and (B) fMRI data processing pipeline. (A) Based on the cytoarchitectonic maximum probability map and macrolabel atlas, the whole brain was parcellated into 212 regions of interest (ROIs), including 142 cortical, 36 subcortical, and 34 cerebellar regions. (B) Voxel-averaged mean time series were extracted from each ROI, and Pearson’s correlation coefficients were calculated for each pair of regions giving rise to 20 correlation matrices for speech production (one for each subject). Six subjects showed inconsistencies during the resting state scan and were removed from further analysis. In a nodal elimination strategy applied to the remaining 14 SPNs, 62 regions were removed from the initial brain parcellation, leaving 150 brain regions for further analysis. The matrices were recomputed and thresholded to obtain a common density range of 77%–86% (10 values, 1% increments). Over this range, group-averaged networks were computed with a density range of 60%–78% (10 values, 2% increments). The sagittal and axial brain views illustrate the relative locations of removed nodes (blue spheres) versus retained nodes (light brown). The same analysis pipeline was applied to the RSN and SylPN. All connectivity matrices are publicly available at http://figshare.com/articles/The_Functional_Connectome_of_Speech_Control/1431873; the codes used to transform the fMRI data to networks can be found at http://research.mssm.edu/simonyanlab/analytical-tools/.

Based on the cytoarchitectonic maximum probability maps and macrolabel atlas [91], the whole brain was parcellated into 212 regions of interest (ROIs), including 142 cortical, 36 subcortical, and 34 cerebellar regions (Fig 8A). For each ROI, a voxelwise-averaged time series of rs-fMRI and task-production fMRI was computed. Because the main focus of this study was to investigate statistical dependence of neural processing sites distributed throughout the entire brain, zero-lag Pearson’s correlation coefficients were calculated for each pair of regions and each condition, giving rise to 212 x 212 correlation matrices (Fig 8B). Visual inspection of per-subject correlation histograms revealed only a negligible number of negative entries, which were removed from the matrices [92,93]. All connectivity matrices are publicly available at http://figshare.com/articles/The_Functional_Connectome_of_Speech_Control/1431873; the codes used to transform the fMRI data to networks can be found at http://research.mssm.edu/simonyanlab/analytical-tools/.

Visual inspection of rs-fMRI data in six subjects showed pronounced atypical variations in average correlation strength, which were suggestive of susceptibility artifacts in the resting-state data. These subjects were excluded from rs-fMRI analysis, and their speech-related fMRI datasets were removed for consistency of data analysis, thus reducing the number of subjects to 14 per group in each condition. None of these six subjects were among the recruited for the follow-up syllable production study.

As a next step, the individual datasets during each condition (i.e., rest, speech, syllable production) were used to construct weighted undirected graphs by using the 212 brain regions as nodes *v*_*i*_ of a network with the associated correlation coefficients representing the weights of the graph's edges. The density (or cost) of each network was computed as the number of actual connections divided by the number of maximal possible connections in the graph [93], which yielded an average density of 88% ± 4% (mean ± SD) for RSN, 92% ± 6% for SPN, and 92% ± 5% for SylPN. Because networks with a density > 50% tend to exhibit random network characteristics [93,94], we reduced the cost of the network by removing edges with a weight less than a given percentage of the maximum weight in the network until the network had a density of 50%. Because our principal goal was to examine the organization of the SPN in comparison to the RSN and SylPN, we first applied this thresholding to the SPN and then adjusted the RSN and SylPN accordingly so that all graphs had the same number of nodes and edges for between-network comparisons [95]. Specifically, the least densely interconnected nodes were first removed from the 212-node SPN, and then the same nodes were excluded from the RSN and SylPN. Such an elimination strategy allowed us to create a refinement of the initial whole-brain parcellation based on the speech production task.

We tested the validity and efficacy of the employed nodal elimination strategy by means of random networks. As a first step, synthetic reference networks with the same number of nonzero elements as the original 212-node per-subject networks were constructed. This gave rise to three groups corresponding to the RSN, SPN, and SylPN with 14 random networks per group. Then the randomized SPN group was thresholded down to 50% density, and the same nodes were then removed from the randomized RSN and SylPN graphs. The resulting downsized networks exhibited a connectivity structure comparable to the initial 212-node random graphs, demonstrating that the employed elimination strategy did not diminish the underlying topological structure of the networks (see details in Methods of S1 Text and S2 Fig).

As a last step in network construction, we removed sparsely connected, low-degree nodes with fewer connections than 5% of the maximal number of nodal links in the speech graphs. Based on this multistep thresholding, a total of 62 nodes were detached from the SPN, which reduced the number of regions from 212 to 150 (73 in the left hemisphere and 77 in the right hemisphere) at a target density of 50% (Fig 8B). The same 62 nodes were then removed from the RSN and SylPN in order to allow for comparisons with SPN properties. The majority of removed regions were located in the ventral parts of the brain, comprising areas that are especially prone to fMRI susceptibility artifacts [96]. All resultant 42 (14 RSN, 14 SPN, and 14 SylPN) weighted undirected graphs with *n* = 150 nodes were thresholded again to obtain a common density range of 77%–86% (10 values, 1% increments). Over this range, group-averaged networks were computed as reported earlier [53] and had a density of 60%–78% (10 values, 2% increments) (Fig 8B).

In the follow-up second experiment, we assessed the global reconfiguration of brain networks during speech production in contrast to the resting state, syllable production, sequential finger tapping, and an auditory discrimination task. The same 212 ROI whole-brain parcellation was used to construct weighted, undirected networks for all experimental conditions, where sparsely-occurring negative correlation values were assigned the edge weight zero [92,93]. In order to avoid a restriction of nodal community formation to an SPN-based subset of nodes, the above-employed nodal elimination strategy was replaced by individual thresholding of the RSN, SylPN, FTN, and ADN independently from the SPN. To ensure comparability of results between different networks and to the findings from the first experiment, all networks were downsized to 150 nodes.

### Graph Theoretical Analysis

#### Experiment 1

We computed four graph metrics, including the degree, strength, clustering coefficient, and efficiency, for each RSN, SylPN and SPN. All computations were performed in Matlab 8.1 [97] using the Brain Connectivity Toolbox [95]. Graph metrics were tested for statistical significance between the SPN and RSN to examine changes in network topology from the resting state to speech production as well as between the SPN and SylPN to quantify the differences between speaking as a complex behavior and syllable production as an isolated motor element of speaking (i.e., motor subnetwork of the SPN). Statistical significance was assessed using a paired two-sample permutation test at an FWE-corrected *p* ≤ 0.05 based on the maximal statistic *T*_max_ [81]_._

To measure the functional influence of a single node, *v*_*i*_, we computed the nodal degree, *k*_*i*_ (i.e., the number of edges connected to the node *v*_*i*_) and its weighted version, the nodal strength, *s*_*i*_ (i.e., the sum of link weights connected to the node *v*_*i*_) [95,98]. Both degree and strength were normalized to the range [0,^1^].

A locally segregated network is known to contain distinct units for specific task processing, thus segregation metrics attempt to quantify a network's predilection for specialized processing. As a first approximation to estimate segregation of the RSN, SPN, and SylPN, we computed the local clustering coefficient *c*_*i*_ of a node *v*_*i*_, which was calculated as the geometric mean of weights in triangles around the node *v*_*i*_ [99]. Local clustering coefficients were compared to null hypothesis networks of random topology, i.e., clustering coefficients were normalized relative to 100 comparable (i.e., degree-, weight-, and strength-distribution preserving) random graphs. As a second step, we analyzed hub formation in the networks. We examined hubs based on a common definition of a network node, *v*_*i*,_ being a hub if *k*_*i*_ is at least one standard deviation greater than the average nodal degree as proposed earlier [51,100]. Similarly, we examined the formation of hubs based on nodal strength.

Most graph measures of integration are based on paths between nodes to allow for quantification of the network's capability for system-wide coordination and coherent states. To estimate network integration, we computed each graph’s global efficiency *E*_*glob*_, which was computed as the average inverse shortest path length in the network [101]. Global efficiency was normalized by 100 corresponding values of random networks. It should be noted that functional networks derived from pairwise cross-correlations represent not only one-to-one connections but also all indirect couplings between the nodes. Thus, path-based metrics, such as efficiency, may yield ambiguous results for correlation-based networks and should be considered with caution [102,103].

Finally, we quantified small-worldness of the networks by the small world index σ, which was computed as the ratio of normalized global clustering coefficient to inverse normalized global efficiency [56].

#### Experiment 2

To assess the extent of network reconfiguration across conditions, we estimated the optimal modular decomposition of the RSN, SPN, and SylPN, as well as the ADN and FTN as control tasks. All networks were reduced to the same size of 150 nodes to ensure comparability to the results from Experiment 1; however, nodal elimination was not based on a SPN-based subset of nodes to avoid introducing a bias in the formation of network communities. An optimal modular decomposition partitions a network into communities by maximizing the number of within-group edges while simultaneously minimizing the number of between-group links [104]. We used a heuristic modularity maximization strategy based on the Kernighan-Lin algorithm [105] to iteratively refine an initial artificial community structure, in which each node formed a separate module. To account for the stochastic nature of the employed optimization routine, which randomly permuted nodal community assignments, the initial network partition was iteratively refined 100 times per network to ensure robustness of the final modular decomposition [106]. Thus, the community refinement strategy was initialized with the artificial community affiliation vector *M* ^0^ = (1,…,*N*) yielding an updated partition vector *M* ^1^, which was used as the initial condition for a second run of the optimization routine, resulting in an updated assignment *M* ^2^, etc. The final community affiliation was defined as the average network partition based on how many times a node was assigned to the same module, where node #1 was used as a reference to account for random module numbering such that node #1 was always assigned to module #1 with neighboring modules numbered consecutively.

To quantify similarity of modular decompositions, we estimated their partition distance, *p*_*d*,_ by calculating the normalized mutual information [107] between the previously computed community affiliation vectors. Based on the determined community structure, we analyzed hub formation in the RSN, SylPN, and SPN compared to the ADN and FTN with respect to each hub’s share of inter- versus intramodular edges, which was quantified by calculating the participation coefficient *pc*_*i*_ [53]. The maximum value of *pc*_*i*_ in a network with *m ≥* 2 modules is 1 –(1 / *m*). All hubs with a *pc*_*i*_ within 10% of this maximum value were classified as connector hubs (linking modules), while hubs with a lower *pc*_*i*_ value were defined as provincial hubs (connecting nodes within the same community).

## Supporting Information

S1 Fig Global clustering coefficient (A) and efficiency (B) of the group-averaged networks across the examined density range of 60%–78%. Solid lines represent RSN (blue), SylPN (green), and SPN (red) values; dashed lines depict the corresponding values of comparable random networks. If not visible, the dashed lines are covered by the solid lines. Normalized values are obtained by dividing RSN, SylPN, or SPN values by the corresponding random network values. All connectivity matrices are publicly available at http://figshare.com/articles/The_Functional_Connectome_of_Speech_Control/1431873; the codes used to transform the fMRI data to networks can be found at http://research.mssm.edu/simonyanlab/analytical-tools/.
(TIF)

S2 Fig High-strength hubs shared by the group-averaged 212-node RSN and SPN and SPN and SylPN. (I) Bar charts show strength values of the top 30% strongest nodes in both the RSN and SPN. Blue bars highlight nodes that are strength-hubs in both RSN and SylPN. (II) Bar charts of the same format show shared strength-hubs of SPN and SylPN among the top 30% strongest nodes. The table shows values of nodal strength with bold numbers indicating hubs. Abbreviations: 1 = area 1; 2 = area 2; 3a = area 3a; 3b = area 3b; 4a = area 4a; 4p = area 4p; 5M = area 5M; 6 = area 6; 7A = area 7A; MTG = middle temporal gyrus; PCu = precuneus. All connectivity matrices are publicly available at http://figshare.com/articles/The_Functional_Connectome_of_Speech_Control/1431873; the codes used to transform the fMRI data to networks can be found at http://research.mssm.edu/simonyanlab/analytical-tools/.
(TIF)

S1 Text Further details regarding graph metrics of the group-averaged networks across all observed density levels are given in Results of S1 Text. An in-depth discussion of the role of subcortical regions for the RSN, SPN, and SylPN in Experiment 1 can be found in Discussion of S1 Text. A detailed analysis of the validity and efficacy of the employed nodal elimination strategy is presented in Methods of S1 Text.
(DOC)

## Abbreviations:

ADN: auditory discrimination network
fMRI: functional MRI
FTN: finger-tapping network
RSN: resting state network
SD: standard deviation
SPN: speech production network
SylPN: syllable production network
