# Supplementary figures and images for: The Functional Connectome of Speech Control
Source: PLoS Biol. 2015 Jul 23;13(7):e1002209. doi: 10.1371/journal.pbio.1002209 (PMC4512708; doi:10.1371/journal.pbio.1002209)

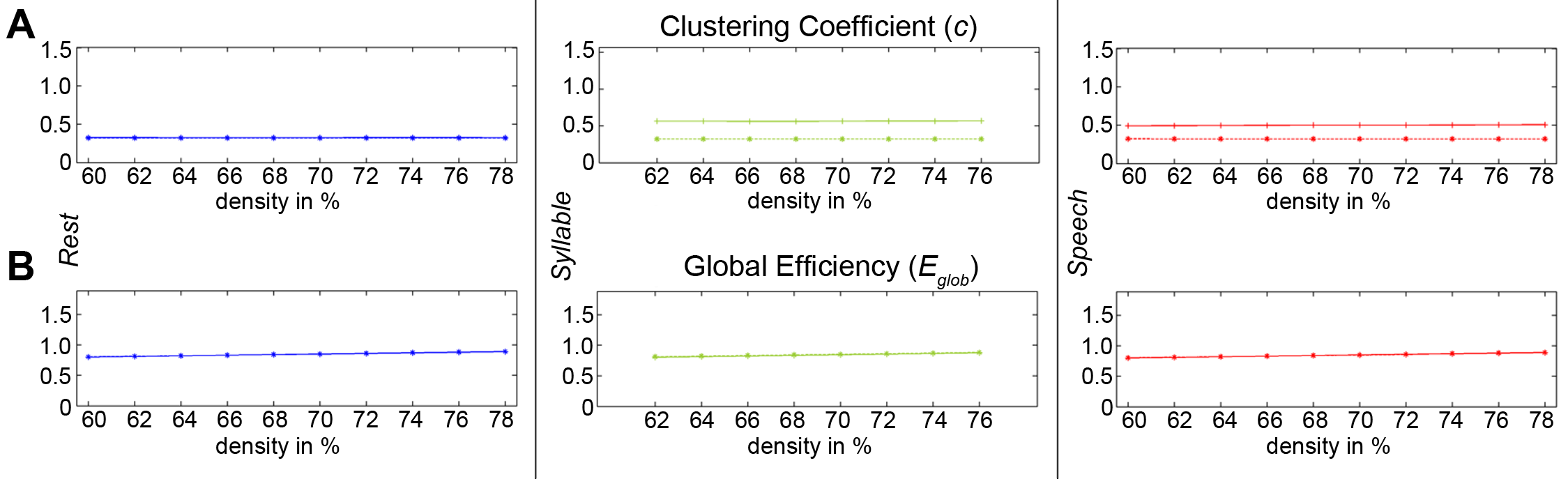

Supplement: S1 Fig — Solid lines represent RSN (blue), SylPN (green), and SPN (red) values; dashed lines depict the corresponding values of comparable random networks. If not visible, the dashed lines are covered by the solid lines. Normalized values are obtained by dividing RSN, SylPN, or SPN values by the corresponding random network values. All connectivity matrices are publicly available at http://figshare.com/articles/The_Functional_Connectome_of_Speech_Control/1431873; the codes used to transform the fMRI data to networks can be found at http://research.mssm.edu/simonyanlab/analytical-tools/. (TIF) [file pbio.1002209.s001.tif]

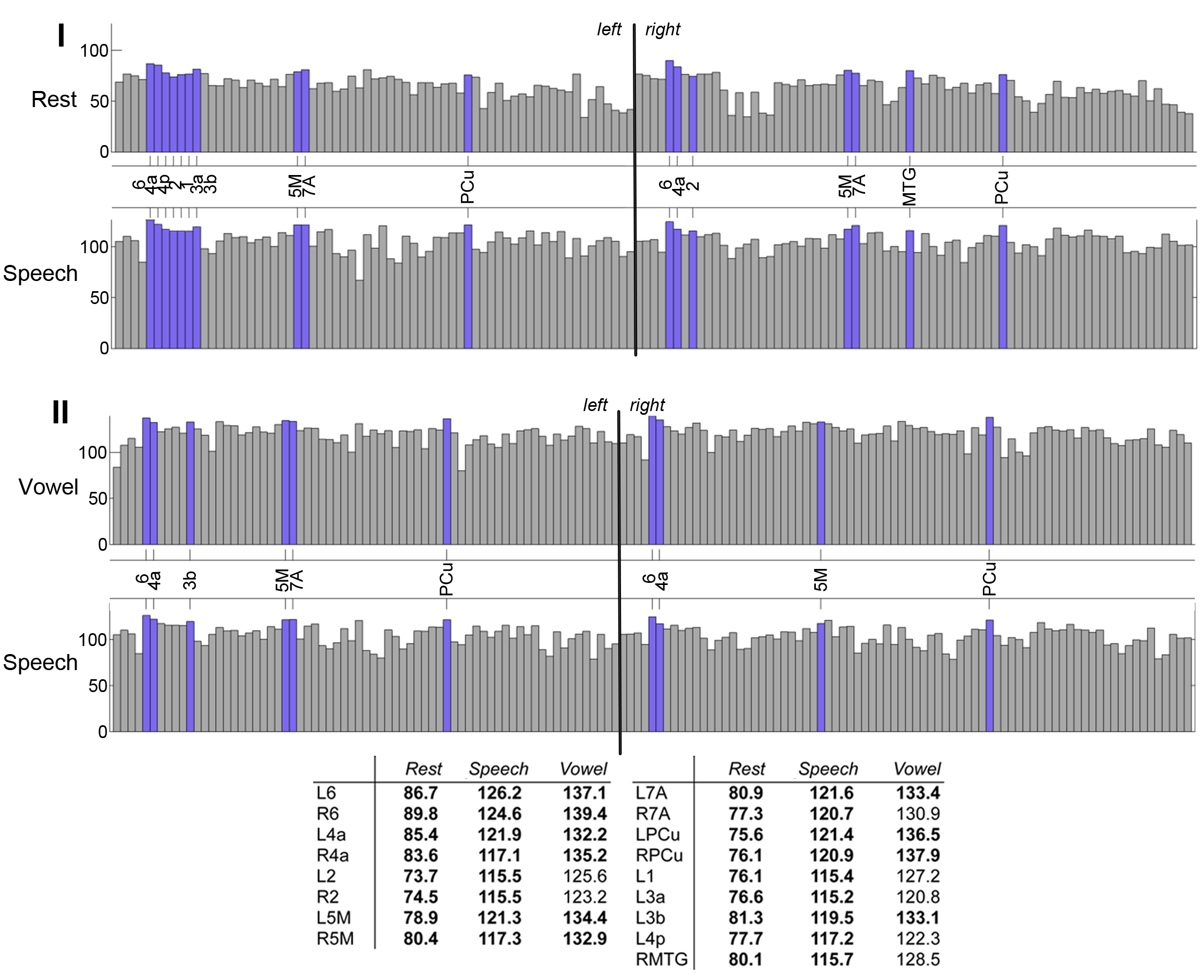

Supplement: S2 Fig — (I) Bar charts show strength values of the top 30% strongest nodes in both the RSN and SPN. Blue bars highlight nodes that are strength-hubs in both RSN and SylPN. (II) Bar charts of the same format show shared strength-hubs of SPN and SylPN among the top 30% strongest nodes. The table shows values of nodal strength with bold numbers indicating hubs. Abbreviations: 1 = area 1; 2 = area 2; 3a = area 3a; 3b = area 3b; 4a = area 4a; 4p = area 4p; 5M = area 5M; 6 = area 6; 7A = area 7A; MTG = middle temporal gyrus; PCu = precuneus. All connectivity matrices are publicly available at http://figshare.com/articles/The_Functional_Connectome_of_Speech_Control/1431873; the codes used to transform the fMRI data to networks can be found at http://research.mssm.edu/simonyanlab/analytical-tools/. (TIF) [file pbio.1002209.s002.tif]
