# Supplementary material for: The Functional Connectome of Speech Control
Source: PLoS Biol. 2015 Jul 23;13(7):e1002209. doi: 10.1371/journal.pbio.1002209 (PMC4512708; doi:10.1371/journal.pbio.1002209)
Supplement: S1 Text — An in-depth discussion of the role of subcortical regions for the RSN, SPN, and SylPN in Experiment 1 can be found in Discussion of S1 Text. A detailed analysis of the validity and efficacy of the employed nodal elimination strategy is presented in Methods of S1 Text. (DOC) [file pbio.1002209.s003.doc]

**The Functional Connectome of Speech Control**

Stefan Fuertinger^1^, Barry Horwitz^2^, Kristina Simonyan^1,3*^

Departments of ^1^Neurology and ^3^Otolaryngology, Icahn School of Medicine at Mount Sinai, New York, NY 10128, ^2^Brain Imaging and Modeling Section, National Institute on Deafness and Other Communication Disorders, National Institutes of Health, Bethesda, MD 20892

**Supporting Information**

**Results**

Global clustering coefficient and global efficiency of the group-averaged RSN, SylPN, and SPN did not exhibit large variations across the considered connection densities confirming the topological stability of the networks independent from thresholding levels (S1 Fig.).

**Discussion**

*The connectivity profile of SPN hub network*

In addition to distinct topological involvement of the prefrontal cortex in the SPN, another important difference between SPN and SylPN (as well as RSN) was the formation of densely connected nodes in the insula, putamen and thalamus (prefrontal, sensorimotor, temporal subdivisions). The functional importance of these brain regions in speech control is not fully understood as ongoing research continuously expands their contribution from an initially suggested role in speech motor planning to associations with higher cognitive processes (for review, see ). The insula is known to establish direct connections with the laryngeal/orofacial sensorimotor cortex, Broca’s area, primary and secondary auditory cortex, and limbic structures, such as anterior cingulate cortices, orbital cortex and amygdala . Recent studies have shown that the insula is involved not only in articulatory planning but also in syntactic and motor processing of complex and novel utterances, increased phonological interference, temporal processing of auditory stimuli, and speech-related control of respiratory activity . A recent study using dynamic causal modeling showed that the insula is positioned serially between Broca’s area (IFG, area 44) and the basal ganglia/cerebellum, from which information is transferred to the premotor and primary motor cortices . Such positioning of the insula may not be accidental as the putamen (along with the IFG) was another brain structure prominently recruited in the SPN but not SylPN.

The basal ganglia, and particularly the putamen, have been considered to be involved in the control of learned voice production (e.g., speech and song) but not innate vocalizations (e.g., human laughing and crying, monkey calls) based on initial clinical evidence that striatal lesions cause different speech disturbances but have essentially no effects on monkey vocalizations . In addition to the involvement of the putamen in the control of speech initiation and motor output, this region has been shown to have associations with syntactic and semantic processing, explicit identification of linguistic aspects of emotional speech prosody, verbal semantic and episodic memory, as well as vocal imitation of novel speech sequences, including learning of a second language . Our results indicate that the putamen plays an important role in the control of real-life speech production but is of less importance for separate speech elements, such as syllables, or the resting state.

Lastly, the thalamic nuclei in the prefrontal, sensorimotor, and temporal subdivisions were strongly correlated nodes within the SPN but not SylPN or RSN. As a major output structure of the basal ganglia and cerebellum and the major subcortical input structure to the corresponding cortical regions, the involvement of these particular thalamic divisions reflects the importance of integration between cortical and subcortical structures for proper organization and balancing of information transfer within the SPN. On the other hand, cortico-subcortical interactions appear overall to be less influential on network topological organization during syllable production and resting.

It is important to note that we confirmed previous findings of RSN involving brain regions necessary for the maintenance of different components of the resting state functional architecture, such as the default mode, visual and auditory resting networks (Fig. 4A). Moreover, we showed that the SylPN, too, retained a distinctive nodal involvement, including the parietal cortex for speech motor learning, monitoring and correcting auditory errors and the cerebellum for timing and automatization of precise motor commands .

**Methods**

*Nodal characteristics of the full 212-node networks*

To ensure robustness of our results with respect to the employed node elimination strategy, we analyzed the connectivity structure of the full 212-node networks and compared the results to the 150-node graphs, which had undergone the density-based node elimination. Similar to the analysis of 150-node networks, we computed shared hubs for the group-averaged 212-node RSN, SPN, and SylPN and analyzed variations of graph metrics in these hub regions in the corresponding per-subject networks across the respective groups. Statistical significance of between-group differences was determined using a paired two-sample permutation test at *p* ≤ 0.05 adjusted for family wise error (FWE) based on the maximal statistic *T*_max_.

*Nodal characteristics of the full 212-node SPN and RSN*

Within the 30% strongest nodes (normalized *s_i_* ≥ 0.7) in SPN and RSN (total of 139 nodes), both networks shared 17 high-strength hubs, including the premotor cortex (bilateral area 6), primary motor cortex (bilateral area 4a and left area 4p), primary somatosensory cortex (bilateral area 2, left areas 3a, 3b and 1), parietal cortex (bilateral areas 5M and 7A), the right middle temporal gyrus and the bilateral precuneus (S2 Fig. (I)). Hence, all high-strength hubs found in the downsized 150-node networks were also present as hub regions in the 212-node graphs. Furthermore, similar to the downsized networks, strength values of the 212-node graphs were significantly higher for all SPN hubs compared to the RSN hubs (all *p* ≤ 0.047).

The analysis of the top 30% most interconnected nodes (normalized *k_i_* ≥ 0.7) in SPN and RSN (total of 193 nodes) revealed no high-degree hubs in RSN or SPN. The high density of the graphs (88±4% (mean±SD) for RSN, 92±6% for SPN) caused the average nodal degree in the networks to be very large, which prohibited the existence of nodes with significantly more connections than all other nodes in the graph. Hence, the identification of high-degree hubs was not possible in the densely connected 212-node RSN or SPN.

*Nodal characteristics of the full 212-node SylPN and SPN*

Among the 30% strongest nodes (normalized *s_i_* ≥ 0.7) in SPN and SylPN (total of 147 nodes), both networks shared 10 high-strength hubs in the premotor cortex (bilateral area 6), primary motor cortex (bilateral area 4a), primary somatosensory cortex (left area 3b), parietal cortex (bilateral area 5M and left area 7A), and the bilateral precuneus (S2 Fig. (II)). These hubs were similar to those found in the 150-node networks, with the only exception that the left lingual gyrus was shared by the 150-node networks only and the right area 5M was shared by the 212-node networks only. However, a closer inspection of network-wide strength values revealed that the left lingual gyrus was a hub for the 212-node SPN only, but not for the 212-node SylPN (where it was still within the top 10% strongest nodes). Conversely, the right area 5M was not a shared hub for the 150 node networks but it was still found among the 30% strongest nodes (compare to Fig. 2-III). Thus, the differences between the 212-node and 150-node networks in terms of hub formation were minimal, if any.

Due to the high density of the 212-node networks (92±5% for SylPN, 92±6% for SPN), the average degree in the graphs was again too large for any degree hubs to emerge among the top 30% most interconnected nodes (normalized *k_i_* ≥ 0.7) in SPN and SylPN (total of 192 nodes).

Thus, the employed nodal elimination strategy helped to emphasize differences between the considered networks while simultaneously conserving the underlying fundamental topological structure of the graphs. As demonstrated above, the high density of the full unthresholded 212-node networks made the identification and meaningful analysis of connection patterns of high-degree hubs impossible. Moreover, the abundance of edges in the 212-node networks also deteriorated the notion of paths in these graphs since the shortest path between most nodes was given by their connecting edge. This made the use of path-based metrics like efficiency and betweenness centrality unfeasible, which provided yet another justification for the design of the proposed nodal elimination strategy.

*Nodal elimination in synthetic networks*

To demonstrate the reliability of the proposed nodal elimination strategy, a numerical experiment with random graphs was conducted. Based on the original SPN, RSN and SylPN weight matrices, synthetic networks were constructed in the following manner: for each per-subject connectivity matrix, a symmetric matrix with the same number of non-zero elements was constructed. Each non-zero entry of the new matrix was a random number drawn from a uniform distribution on the half-open interval (0,1]. This gave rise to 42 (based on 14 RSN, 14 SPN, 14 SylPN) symmetric random matrices, which served as weight matrices of undirected random networks, from now on denoted by rRSN, rSPN and rSylPN, respectively. By construction, rRSN, rSPN, and rSylPN had the same density as the corresponding empirical RSN, SPN, and SylPN, but the locations and weights of edges were randomized.

Following the analysis of the empirical networks, differences in the topological structure of rRSN, rSPN, and rSylPN were analyzed in terms of nodal degree, strength, and hub formation in the group-averaged networks. Statistical significance between comparisons was assessed using a paired two-sample permutation test at *p* ≤ 0.05 adjusted for family wise error (FWE) based on the maximal statistic *T*_max_.

While all strength-hubs in the full 212-node RSN and SPN showed a significant difference in strength, the strength values in only one hub differed between rRSN and rSPN (*p* = 0.02). Unlike the empirical 212-node networks, which did not admit any degree hubs, rRSN and rSPN shared six degree hubs, two of which showed a significant difference in *k_i_* (*p* ≤ 0.01). In contrast, none of the shared degree/strength hubs in rSPN and rSYLPN exhibited any statistical differences in values (all *p* > 0.89). Thus, while having the same number of edges as the empirical networks, the random graphs did not reveal similar topological features.

As a next step the proposed nodal elimination strategy was tested on the constructed random networks. First, rSPN was thresholded down to 50% connection density, disconnecting 128 nodes in the process, then the same nodes were removed from rRSN and rSylPN, and the networks were tested for differences in nodal strength, degree, and hub formation. The downsized networks did not reveal a change in topological structure. Group-averaged rSPN and rSylPN shared five strength/degree hubs, which did not show any statistical difference in strength/degree values between the respective groups (all *p* ≥ 0.63). Similarly, six shared hubs were found for rRSN and rSPN, none of which exhibited a pronounced difference in values between the two groups (all *p* ≥ 0.07).

To guarantee independence of this result from the choice of the elimination basis, the above experiment was repeated with rSylPN and rRSN in place of rSPN. Thus, rSylPN was thresholded down to 50% density, thus disconnecting 100 nodes, which were then also removed from rSPN and rRSN. None of a total of eight shared hubs showed a significant difference in strength/degree between rSylPN and rSPN (all *p* ≥ 0.35), the values of two of overall six degree hubs shared by rSylPN and rRSN differed (*p* ≤ 0.04). Similarly, when eliminating nodes based on thresholding rRSN to 50% density (removing 110 nodes), none of the hubs shared by rRSN and rSylPN showed any variation in degree/strength (all *p* ≥ 0.06), and only one of two degree-hubs common to both rRSN and rSPN exhibited statistically different values between the groups (*p* = 0.045).

It should be noted that the considered random graphs were constructed based on the number of edges in the empirical per-subject networks. The lower number of links in the empirical RSN as compared to SPN/SylPN (which showed a comparable number of edges) may account for the fact that rRSN/rSPN as well as rRSN/rSylPN showed some statistical differences with respect to *k_i_*/*s_i_* values at a few hub regions, while this was not observed for rSPN/rSylPN. This randomization bias was intentionally introduced in the design of this numerical experiment to further emphasize the reliability of the proposed nodal elimination strategy. Although the random graphs were based on empirical networks and despite the fact that a considerable number of nodes were removed (up to 128 of 212 vertices, around 60%) the proposed nodal elimination strategy did not introduce an artificial change in the connectivity structure of the networks.

**References**

1. Price CJ (2012) A review and synthesis of the first 20 years of PET and fMRI studies of heard speech, spoken language and reading. Neuroimage 62: 816-847.

2. Simonyan K, Jurgens U (2002) Cortico-cortical projections of the motorcortical larynx area in the rhesus monkey. Brain Res 949: 23-31.

3. Simonyan K, Jurgens U (2005) Afferent cortical connections of the motor cortical larynx area in the rhesus monkey. Neuroscience 130: 133-149.

4. Cauda F, D'Agata F, Sacco K, Duca S, Geminiani G, et al. (2011) Functional connectivity of the insula in the resting brain. Neuroimage 55: 8-23.

5. Moro A, Tettamanti M, Perani D, Donati C, Cappa SF, et al. (2001) Syntax and the brain: disentangling grammar by selective anomalies. Neuroimage 13: 110-118.

6. Wise RJ, Greene J, Buchel C, Scott SK (1999) Brain regions involved in articulation. Lancet 353: 1057-1061.

7. Dronkers NF (1996) A new brain region for coordinating speech articulation. Nature 384: 159-161.

8. Riecker A, Ackermann H, Wildgruber D, Meyer J, Dogil G, et al. (2000) Articulatory/phonetic sequencing at the level of the anterior perisylvian cortex: a functional magnetic resonance imaging (fMRI) study. Brain Lang 75: 259-276.

9. Mechelli A, Josephs O, Lambon Ralph MA, McClelland JL, Price CJ (2007) Dissociating stimulus-driven semantic and phonological effect during reading and naming. Hum Brain Mapp 28: 205-217.

10. Ackermann H, Riecker A (2004) The contribution of the insula to motor aspects of speech production: a review and a hypothesis. Brain Lang 89: 320-328.

11. Shuster LI, Lemieux SK (2005) An fMRI investigation of covertly and overtly produced mono- and multisyllabic words. Brain Lang 93: 20-31.

12. Shuster LI (2009) The effect of sublexical and lexical frequency on speech production: An fMRI investigation. Brain Lang 111: 66-72.

13. Ackermann H, Riecker A (2010) The contribution(s) of the insula to speech production: a review of the clinical and functional imaging literature. Brain Struct Funct 214: 419-433.

14. Loucks TM, Poletto CJ, Simonyan K, Reynolds CL, Ludlow CL (2007) Human brain activation during phonation and exhalation: common volitional control for two upper airway functions. Neuroimage 36: 131-143.

15. Eickhoff SB, Heim S, Zilles K, Amunts K (2009) A systems perspective on the effective connectivity of overt speech production. Philos Trans A Math Phys Eng Sci 367: 2399-2421.

16. Cummings JL (1993) Frontal-subcortical circuits and human behavior. Arch Neurol 50: 873-880.

17. Damasio AR, Damasio H, Rizzo M, Varney N, Gersh F (1982) Aphasia with nonhemorrhagic lesions in the basal ganglia and internal capsule. Arch Neurol 39: 15-24.

18. Jurgens U, Kirzinger A, von Cramon D (1982) The effects of deep-reaching lesions in the cortical face area on phonation. A combined case report and experimental monkey study. Cortex 18: 125-139.

19. Lee MS, Lee SB, Kim WC (1996) Spasmodic dysphonia associated with a left ventrolateral putaminal lesion. Neurology 47: 827-828.

20. Nadeau SE, Crosson B (1997) Subcortical aphasia. Brain Lang 58: 355-402; discussion 418-323.

21. Watkins KE, Dronkers NF, Vargha-Khadem F (2002) Behavioural analysis of an inherited speech and language disorder: comparison with acquired aphasia. Brain 125: 452-464.

22. Davis MH, Gaskell MG (2009) A complementary systems account of word learning: neural and behavioural evidence. Philos Trans R Soc Lond B Biol Sci 364: 3773-3800.

23. Kotz SA, Frisch S, von Cramon DY, Friederici AD (2003) Syntactic language processing: ERP lesion data on the role of the basal ganglia. J Int Neuropsychol Soc 9: 1053-1060.

24. Price CJ (2010) The anatomy of language: a review of 100 fMRI studies published in 2009. Ann N Y Acad Sci 1191: 62-88.

25. Bach DR, Grandjean D, Sander D, Herdener M, Strik WK, et al. (2008) The effect of appraisal level on processing of emotional prosody in meaningless speech. Neuroimage 42: 919-927.

26. Koylu B, Trinka E, Ischebeck A, Visani P, Trieb T, et al. (2006) Neural correlates of verbal semantic memory in patients with temporal lobe epilepsy. Epilepsy Res 72: 178-191.

27. Ystad M, Eichele T, Lundervold AJ, Lundervold A (2010) Subcortical functional connectivity and verbal episodic memory in healthy elderly--a resting state fMRI study. Neuroimage 52: 379-388.

28. Klein D, Milner B, Zatorre RJ, Meyer E, Evans AC (1995) The neural substrates underlying word generation: a bilingual functional-imaging study. Proc Natl Acad Sci U S A 92: 2899-2903.

29. Klein D, Zatorre RJ, Milner B, Meyer E, Evans AC (1994) Left putaminal activation when speaking a second language: evidence from PET. Neuroreport 5: 2295-2297.

30. Liegeois F, Morgan AT, Connelly A, Vargha-Khadem F (2011) Endophenotypes of FOXP2: dysfunction within the human articulatory network. Eur J Paediatr Neurol 15: 283-288.

31. Smith SM, Fox PT, Miller KL, Glahn DC, Fox PM, et al. (2009) Correspondence of the brain's functional architecture during activation and rest. Proc Natl Acad Sci U S A 106: 13040-13045.

32. Hartwigsen G, Baumgaertner A, Price CJ, Koehnke M, Ulmer S, et al. (2010) Phonological decisions require both the left and right supramarginal gyri. Proc Natl Acad Sci U S A 107: 16494-16499.

33. Kort NS, Nagarajan SS, Houde JF (2014) A bilateral cortical network responds to pitch perturbations in speech feedback. Neuroimage 86: 525-535.

34. Shum M, Shiller DM, Baum SR, Gracco VL (2011) Sensorimotor integration for speech motor learning involves the inferior parietal cortex. Eur J Neurosci 34: 1817-1822.

35. Hickok G, Poeppel D (2007) The cortical organization of speech processing. Nat Rev Neurosci 8: 393-402.

36. Thurling M, Kuper M, Stefanescu R, Maderwald S, Gizewski ER, et al. (2011) Activation of the dentate nucleus in a verb generation task: A 7T MRI study. Neuroimage 57: 1184-1191.

37. Stoodley CJ, Schmahmann JD (2009) Functional topography in the human cerebellum: a meta-analysis of neuroimaging studies. Neuroimage 44: 489-501.

38. Stoodley CJ, Schmahmann JD (2010) Evidence for topographic organization in the cerebellum of motor control versus cognitive and affective processing. Cortex 46: 831-844.

39. Bohland JW, Guenther FH (2006) An fMRI investigation of syllable sequence production. Neuroimage 32: 821-841.

40. Guenther FH, Ghosh SS, Tourville JA (2006) Neural modeling and imaging of the cortical interactions underlying syllable production. Brain Lang 96: 280-301.

41. Ben-Yehudah G, Guediche S, Fiez JA (2007) Cerebellar contributions to verbal working memory: beyond cognitive theory. Cerebellum 6: 193-201.
